# Ethnic-specific ZJU index thresholds for hepatic steatosis and fibrosis in Chinese MASLD

**DOI:** 10.3389/fmed.2026.1799299

**Published:** 2026-05-05

**Authors:** Yu Qiu, Zhiqi Zhang, Yichuan Wan, Yueting Zhang, Chenyu Hu, Yi Huang

**Affiliations:** 1 Department of Hepatic Diseases of Chongqing Hospital of Traditional Chinese Medicine, Chongqing, China; 2 College of Traditional Chinese Medicine, Chongqing Medical University, Chongqing, China

**Keywords:** CAP, hepatic steatosis, liver fibrosis, LSM, MASLD, VCTE, ZJU index

## Abstract

**Background:**

The Zhejiang University (ZJU) index correlates with metabolic dysfunction-associated steatotic liver disease (MASLD) in Chinese cohorts, but its ethnic-specific thresholds for hepatic steatosis and fibrosis remain undefined.

**Methods:**

A cross-sectional study of 2,156 Chinese MASLD patients used vibration-controlled transient elastography (VCTE) and restricted cubic spline (RCS) modeling to analyze ZJU index associations with controlled attenuation parameter (CAP) and liver stiffness measurement (LSM).

**Results:**

The ZJU index independently correlated with both controlled attenuation parameter (CAP, β = 0.96 dB/m) and liver stiffness measurement (LSM, β = 0.042 kPa). Crucially, we identified ethnic-specific thresholds for Chinese populations (CAP: 46.38; LSM: 49.49) that are significantly lower than Western cohorts, characterized by rapid pre-threshold risk escalation and post-threshold plateaus. The strongest correlations appeared in males, the elderly, and non-obese individuals. Sensitivity analyses using multiple imputation showed materially consistent results. In addition, comparative analyses indicated that the ZJU index had generally comparable discriminatory performance compared to conventional indicators, outperforming several metabolic markers for fibrosis-related outcomes, although body mass index (BMI) and waist circumference showed slightly higher area under the curve (AUC) values in some fibrosis models.

**Conclusion:**

The ZJU index is a cost-effective tool for Chinese MASLD assessment, with ethnic-specific thresholds critical for accurate disease stratification in resource-limited settings.

## 1 Introduction

Metabolic dysfunction-associated steatotic liver disease (MASLD), which refers to hepatic fat accumulation related to metabolic risk factors such as obesity, diabetes, or hypertension (1–3), is a globally prevalent disease affecting approximately a quarter of the world's population. In China, 15%−20% of adults are affected, creating significant difficulties for healthcare systems (4–8). Systemic metabolic dysfunction is driven by hepatic lipid accumulation (9–13). Its progressive hepatic steatosis and fibrosis significantly increase all-cause mortality (14, 15), so an accurate stage of the disease is needed. However, a liver biopsy remains invasive with poor variability acceptance (16). At the same time, vibration-controlled transient elastography (VCTE) is also restricted in areas with limited resources (17, 18), especially in rural China, where the MASLD rate is rapidly increasing. Therefore, there is an urgent need for simple, low-cost, and widely accessible non-invasive tools to diagnose and stage MASLD, particularly in resource-limited settings.

The Zhejiang University (ZJU) index is a metabolic index calculated from readily available clinical and laboratory variables, including BMI, fasting glucose, triglycerides (TGs), alanine aminotransferase (ALT)/aspartate transaminase (AST) ratio, and sex. It has been developed as a non-invasive screening tool for MASLD detection. Wang et al. (19) first validated the ZJU index for MASLD detection in Chinese populations. Subsequent studies have further demonstrated its superior performance compared to other metabolic indices (19, 20). Luo et al. (21) further established the relationship between the ZJU index and VCTE measurements (CAP and LSM) in a US population. This makes the ZJU index particularly valuable as a cost-effective screening tool in resource-limited settings where liver biopsy and VCTE may be unavailable (16–18).

However, it is problematic to directly apply the thresholds derived from Western populations to Asian populations. Asians exhibit a unique “lean MASLD” phenotype—defined as the presence of MASLD in individuals with a normal body mass index—which is 2–3 times more common in Asia (affecting approximately 30% of normal-weight individuals) compared to Western populations (22–24). The metabolic divergence is caused by genetic factors, with the PNPLA3 rs738409 G allele having more severe hepatotoxicity effects on Asians (OR=1.92, 95% CI: 1.54–2.39) than non-Europeans (OR = 1.73, 95% CI: 1.54–1.95) (22–24), and more visceral fat accumulation at a lower BMI threshold (25). These ethnic metabolic differences necessitate population-specific diagnostic cutoffs. While the ZJU index has demonstrated good diagnostic validity in Western populations, it remains unclear whether Chinese patients with MASLD require ethnicity-specific thresholds for accurate disease assessment.

Therefore, this study aimed to address the significant knowledge gap by validating ZJU index-VCTE correlations in Chinese patients with MASLD and developing population-specific diagnostic thresholds that account for ethnic-specific metabolic phenotypes. This was achieved by providing much-needed diagnostic tools to the world's largest at-risk population, where VCTE and liver biopsy may not be available in resource-limited healthcare settings.

## 2 Methods

### 2.1 Study design and participants

**Figure 1:**
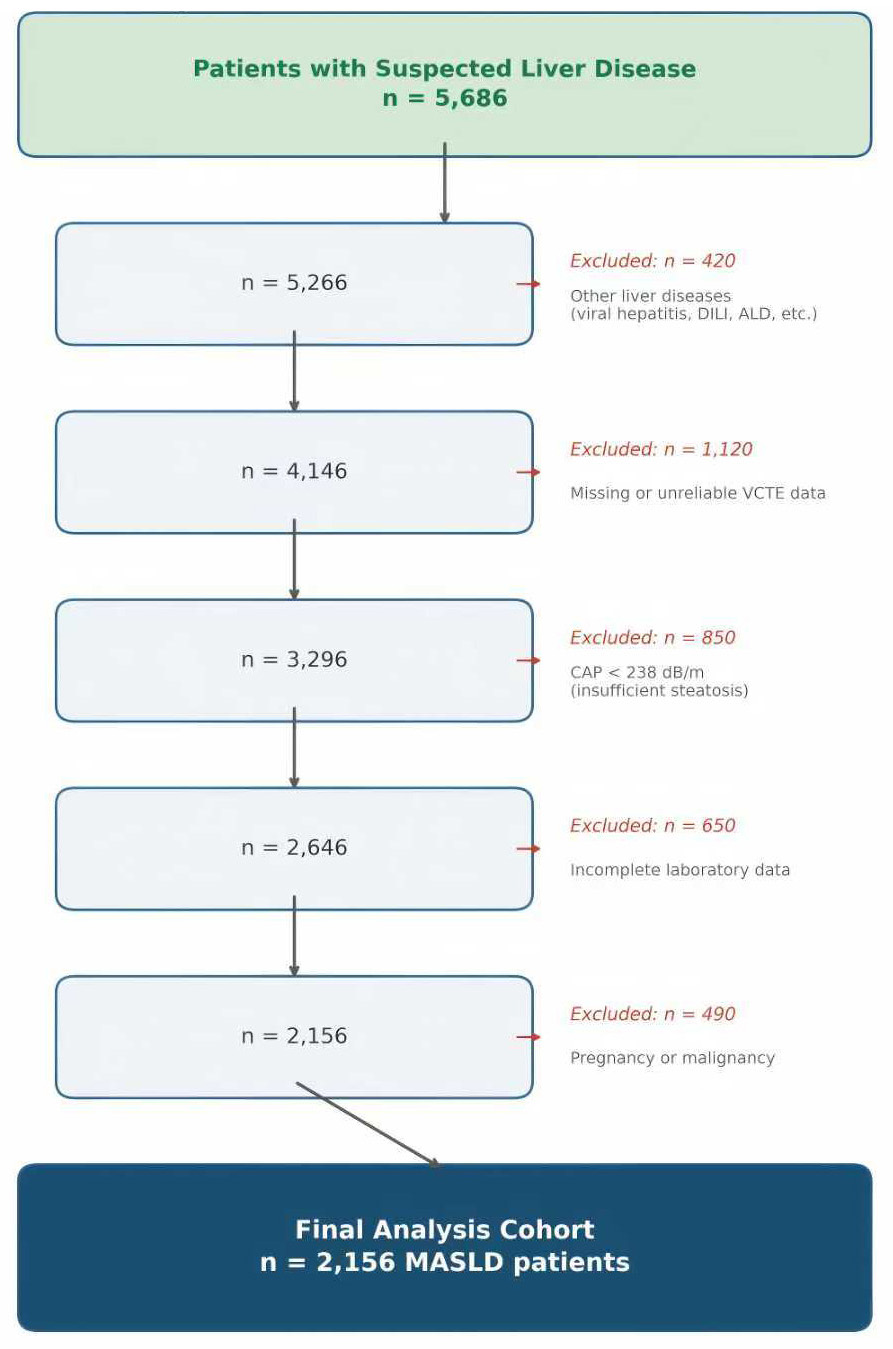
Patient selection flowchart. Study period: 2022-2025. Location: Chongqing Hospital of Traditional Chinese Medicine. VCTE, vibration-controlled transient elastography; CAP, controlled attenuation parameter; MASLD, metabolic dysfunction-associated steatotic liver disease; DILI, drug-induced liver injury; ALD, alcohol-associated liver disease.

This cross-sectional study was conducted in accordance with the Strengthening the Reporting of Observational Studies in Epidemiology (STROBE) guidelines. Initially, we screened 5,686 patients suspected of having liver disease at Chongqing Hospital of Traditional Chinese Medicine (2022–2025). Applying stringent inclusion/exclusion criteria, we ultimately included 2,156 patients with a definitive diagnosis of MASLD in the final analysis (Figure 1). The research protocol was approved by the Ethics Committee of Chongqing Hospital of Traditional Chinese Medicine (No. 2025-IIT-KS-13). According to the requirements of the Chinese ethical review measures for biomedical research involving humans (NHC Order No. 11), which govern the conduct of retrospective studies using anonymous patient data, the requirement for informed consent was waived. Patient information was deidentified before the analysis took place to protect patient privacy.

#### 2.1.1 MASLD diagnosis

MASLD requires the presence of hepatic steatosis alongside one or more cardiometabolic risk criteria while excluding significant alcohol consumption (1, 2). The five cardiometabolic risk factors include (1) BMI ≥24 kg/m^2^ (26) or waist circumference >90 cm (males) or >80 cm (females); (2) impaired fasting glucose, pre-diabetic state, or established type 2 diabetes; (3) blood pressure ≥130/85 mmHg or current antihypertensive therapy; (4) plasma triglycerides ≥1.70 mmol/L; or (5) plasma HDL cholesterol < 1.0 mmol/L (males) or < 1.3 mmol/L (females).

#### 2.1.2 Study population

Initially, 5,686 patients with suspected liver disease were screened. After applying the inclusion and exclusion criteria, 2,156 MASLD patients were included in the final analysis (as shown in Figure 1).

#### 2.1.3 Inclusion criteria

The inclusion criteria included patients with (1) age ≥18 years, (2) confirmed MASLD diagnosis, (3) complete VCTE examination data, and (4) available laboratory parameters for ZJU index calculation.

#### 2.1.4 Exclusion criteria

The exclusion criteria included patients with (1) other liver diseases, including viral hepatitis [hepatitis B surface antigen (HBsAg) or anti-hepatitis C virus (HCV) positive], drug-induced liver injury, autoimmune liver disease, and alcohol-associated liver disease (alcohol consumption >20 g/day for women or >30 g/day for men); (2) missing VCTE data or unreliable measurements; (3) CAP < 238 dB/m (below steatosis threshold) (27); (4) incomplete laboratory data preventing ZJU index calculation; and (5) pregnancy or malignancy.

#### 2.1.5 Multiple imputation and sensitivity analysis

To assess the robustness of the findings and address potential selection bias related to the exclusion of participants with incomplete laboratory data, we performed multiple imputation as a sensitivity analysis. The primary regression analyses were repeated after imputation, and the results were compared with those of the complete-case analysis. Additional sensitivity analyses included bootstrap resampling, alternative model specifications, subgroup analyses, and cross-validation.

### 2.2 ZJU index calculation

The ZJU index was calculated using the validated formula (19):


$$\begin{array}{l} \text{ZJU index}=\text{BMI }\left(\text{kg}/{\text{m}}^{2}\right)\;+\text{ FBG }\left(\text{mmol}/\text{L}\right)\;+\text{ TG }\left(\text{mmol}/\text{L}\right)\;\\ +3\times \left(\text{ALT}/\text{AST ratio}\right)\;+\;2\;\left(\text{if female}\right). \end{array}$$

#### 2.2.1 Comparative analysis of the ZJU index

To evaluate the relative strengths and limitations of the ZJU index, we compared its discriminatory performance with conventional anthropometric and metabolic indicators, including BMI, waist circumference, triglycerides, fasting plasma glucose, ALT/AST ratio, and the triglyceride–glucose (TyG) index. Receiver operating characteristic (ROC) analyses were performed for CAP- and LSM-defined liver outcomes, and pairwise AUC comparisons were conducted using the DeLong test.

### 2.3 VCTE measurements

VCTE evaluations were conducted using the FibroScan 502 V2 Touch (Echosens, Paris, France) by two certified technicians with more than 500 examinations each (inter-operator ICC = 0.912). Participants underwent a minimum 3-h fasting period (verified by patient interview) and were placed in the correct lateral decubitus position with the right arm in maximal abduction. The transducer was placed at the right mid-axillary line in the intercostal space between ribs nine and 11. The quality criteria strictly adhered to the European Association for the Study of the Liver (EASL) 2021 guidelines (18): a minimum of 10 valid measurements (actual mean: 12.3 ± 2.1 per patient), an interquartile range (IQR)/median ratio of < 30% for LSM (exclusion if >30%), a success rate of ≥60% (actual cohort mean: 87.4%), and probe selection based on patient characteristics (M probe: 89.2% of patients; XL probe for obese patients with skin-capsule distance >25 mm: 10.8%). Technicians were blinded to patient clinical data and ZJU index values to prevent measurement bias.

Two metrics were evaluated: LSM (kPa) quantifies liver stiffness as a surrogate for fibrosis stage (normal < 5.0 kPa; significant fibrosis ≥7.0 kPa; advanced fibrosis ≥9.0 kPa) (18), and CAP (dB/m) quantifies ultrasonic attenuation as a marker of hepatic fat content (CAP ≥238 dB/m defines significant steatosis per validation studies) (27).

### 2.4 Covariates and statistical analysis

Covariates were selected based on the literature (28–30). To avoid multicollinearity, we excluded covariates from the ZJU index. The following variables were included: demographic data (age, gender, educational level, employment status, and monthly income); anthropometric data [waist circumference (WC), hip circumference (HC), waist-to-hip ratio (WHR), and body fat percentage (BFP)]; blood test indicators [alanine aminotransferase (ALT), aspartate transaminase (AST), alkaline phosphatase (ALP), total protein (TP), globulin (G), albumin (A), albumin-to-globulin ratio (A/G), γ-glutamyl transferase (GGT), total cholesterol (TC), low-density lipoprotein cholesterol (LDL-C), high-density lipoprotein cholesterol (HDL-C), triglycerides (TG), glucose (GLU), and uric acid (UA)]; and lifestyle habits (dining behavior, taste, number of exercises per week, and daily routines). The variance inflation factors (VIFs) of all the covariates were less than 3.0 (1.14–2.94). Therefore, it can be concluded that there is no problematic collinearity.

The continuous variables were assessed for normality using Shapiro–Wilk tests. ZJU index quartiles were stratified based on participants, and analysis of variance (ANOVA) or Kruskal–Wallis tests were used for continuous variables and chi-squared tests for categorical variables in between-group comparisons. The associations between the ZJU index and VCTE parameters (CAP/LSM) were evaluated by constructing three hierarchical linear regression models with the following adjusted variables included: Model 1 (unadjusted), Model 2 (adjusted for sex, age, and occupational type), and Model 3 (fully adjusted for all the variables).

Non-linear relationships were evaluated via RCS; four knots were put at the 5th, 35th, 65th, and 95th percentiles of the ZJU index distribution. When RCS models detected non-linearity (*P* < 0.05 in the non-linearity test), piecewise linear regression models were fitted using a two-piecewise linear model with automatic identification of the inflection point via a segmented regression analysis using the Davies test to locate the threshold. Model selection was compared across linear and piecewise models with likelihood ratio tests and Akaike information criterion (AIC). Internal validation utilized bootstrap resampling (1,000 iterations for CAP models and 100 iterations for LSM models, due to the computational burden). Each of the iterations involved taking 2,156 patients with replacement, re-estimating the piecewise regression parameters, and calculating bias-corrected 95% CIs via the percentile method. Bootstrap success rates for all models are 100%.

Effect modification was explored across clinically essential subgroups: sex, age (< 45, 45–65, and ≥65 years), BMI (< 30 and ≥30 kg/m^2^), job type, how often exercise is performed, and taste preference. Multiplicative interaction terms were tested for heterogeneity (*P*-interaction < 0.10). Statistical significance was set at an alpha level of 0.10 rather than 0.05 for interaction terms; this is a widely adopted standard in epidemiological studies because interaction tests are typically underpowered, and a less stringent threshold helps mitigate the risk of Type II errors when detecting significant clinical effect modifications. All analyses were run using R version 4.4.1 (R Foundation for Statistical Computing, Vienna, Austria). The following packages were used: segmented, for threshold detection; rms, for RCS modeling; and boot, for bootstrap validation. Two-sided tests at the α level of 0.05 were conducted to assess significance.

## 3 Results

### 3.1 Baseline characteristics

**Figure 2:**
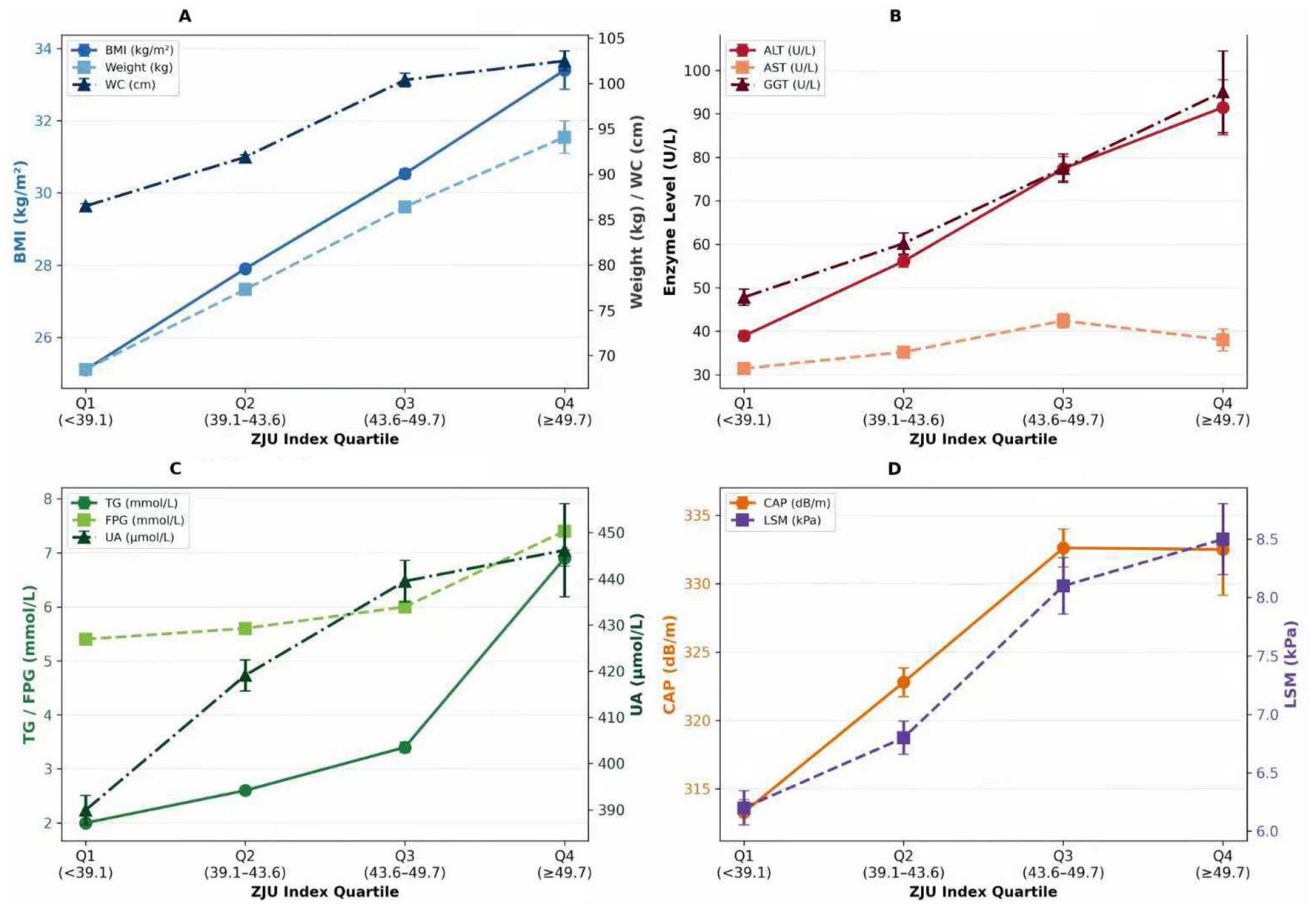
Metabolic profiles across ZJU index quartiles. **(A)** Anthropometric parameter, **(B)** Hepatic enzymes, **(C)** Lipid and glucose metabolism, **(D)** VCTE parameters. Data presented as mean ± SE. All *P* for trend < 0.01 across quartiles (ANOVA). Quartile cutoffs: Q1 (<39.1), Q2 (39.1–43.6), Q3 (43.6–49.7), Q4 (≥49.7). *n* = 2,156. BMI, body mass index; WC, waist circumference; ALT, alanine aminotransferase; AST, aspartate aminotransferase; GGT, gamma-glutamyl transferase; TG, triglycerides; FPG, fasting plasma glucose; UA, uric acid; CAP, controlled attenuation parameter; LSM, liver stiffness measurement.

This observational evaluation encompassed 2,156 Chinese individuals with MASLD (mean age: 44.20 ± 13.97 years; 77.6% male). ZJU index quartiles stratified participants into four categories: Q1 (< 39.10, *n* = 794), Q2 (39.10–43.60, *n* = 791), Q3 (43.60–49.70, *n* = 467), and Q4 (≥49.70, *n* = 104). The metabolic deterioration progressed across the ascending quartiles; all demographic and biochemical parameters showed significant interquartile differences (*P* < 0.001). Q4 had the worst metabolic derangement and high anthropometric measures (BMI: 33.4 ± 5.4 kg/m^2^, WC: 102.5 ± 11.2 cm), as well as elevated hepatic enzymes (ALT: 91.5 ± 64.6 U/L) and lipids (triglycerides: 6.9 ± 1.5 mmol/L). Critically, both CAP (313.3 ± 25.9 dB/m in Q1 vs. 332.5 ± 34.1 dB/m in Q4) and LSM (6.2 ± 4.1 kPa vs. 8.5 ± 3.1 kPa) were substantially higher in the upper ZJU quartiles (Table 1, Figure 2).

**Table 1:** Weighted characteristics of the study population based on ZJU quartiles.

Characteristic	Q1 (< 39.1)	Q2 (39.1–43.6)	Q3 (43.6–49.7)	Q4 (≥49.7)	*P*-value
Number	794	791	467	104	
15.6-7.2,-1.3498ptAge, year	45.8 ± 13.6	43.8 ± 13.8	42.9 ± 14.6	41.3 ± 14.0	< 0.01
Sex, %					
Male	78.5	74.7	81.8	74	< 0.01
15.6-7.2,-1.3498ptFemale	21.5	25.3	18.2	26	
Occupation, %					
Mental effort	49.1	50.2	41.8	40.4	< 0.01
Physical effort	29.4	27	30.8	27.9	
Unemployed	21.5	22.8	27.4	31.7	
Exercise frequency, %					
Frequent	7.3	6.5	3.2	3.9	< 0.01
Moderate	40.1	30.8	26.6	26.9	
Insufficient	52.6	62.7	70.2	69.2	
Income level, %					
Low income	17.8	17.7	16.1	17.3	< 0.01
Lower-middle income	30.4	28.3	29.1	26.9	
Upper-middle income	37.9	38.1	40.5	33.7	
High income	14	15.9	14.3	22.1	
Dietary habits, %					
Picky eating	2.1	1.6	2.1	1	< 0.01
Binge eating	10.3	10.5	18.4	20.2	
Regular eating habits	69.9	60.9	52.7	47.1	
Irregular eating habits	12	19.1	18	19.2	
Frequent late-night snacking	5.7	7.9	8.8	12.5	
Daily routine, %					
Basic routine	76.4	70.7	63.4	54.8	< 0.01
Irregular routine	20	23.8	31.3	37.5	
Nocturnal lifestyle	3.6	5.5	5.3	7.7	
Educational level, %					
Primary education	5	5.7	2.4	1	< 0.01
Middle school education	16.8	14.3	12	12.5	
High school education	22.7	20.9	21.2	19.2	
University education	50	52.1	58.2	63.5	
Postgraduate education	5.5	7.1	6.2	3.8	
Taste preference					
Normal taste preference	29.7	26	21	15.4	< 0.01
Preference for light flavors	11.1	14.5	16.1	23.1	
Preference for greasy and sweet flavors	32.2	37.2	44.1	46.2	
Other preference flavors	27	22.3	18.8	15.3	
Weight, kg	68.5 ± 8.8	77.3 ± 9.7	86.4 ± 11.6	94.1 ± 18.2	< 0.01
BMI, kg/m^2^	25.1 ± 1.8	27.9 ± 2.0	30.53 ± 2.87	33.4 ± 5.4	< 0.01
Waist circumference, cm	86.5 ± 7.6	91.9 ± 7.0	100.4 ± 16.3	102.5 ± 11.2	< 0.01
Abdominal circumference, cm	88.7 ± 6.4	94.1 ± 6.6	99.7 ± 7.6	104.4 ± 12.1	< 0.01
Hip circumference, cm	95.2 ± 5.3	99.6 ± 5.3	103.3 ± 6.2	106.3 ± 13.5	< 0.01
Waist-to-hip ratio (WHR)	0.9 ± 0.1	0.9 ± 0.1	1.0 ± 0.1	1.0 ± 0.1	< 0.01
ALT, U/L	38.9 ± 33.0	56.1 ± 39.5	77.4 ± 60.9	91.5 ± 64.6	< 0.01
AST, U/L	31.4 ± 23.6	35.2 ± 24.6	42.4 ± 36.2	38.0 ± 25.9	< 0.01
GGT, U/L	47.8 ± 53.1	60.2 ± 68.3	77.5 ± 71.1	95.1 ± 95.6	< 0.01
ALP, U/L	78.9 ± 31.3	77.6 ± 20.9	80.4 ± 26.2	85.6 ± 29.9	< 0.01
TG, mmol/L	2.0 ± 1.0	2.6 ± 1.3	3.4 ± 2.1	6.9 ± 1.5	< 0.01
TC, mmol/L	5.0 ± 1.0	5.2 ± 1.0	5.2 ± 1.0	5.5 ± 1.2	< 0.01
HDL-C, mmol/L	1.2 ± 0.4	1.2 ± 0.4	1.1 ± 0.5	1.2 ± 0.7	< 0.01
LDL-C, mmol/L	3.2 ± 1.0	3.3 ± 1.0	3.2 ± 1.0	3.2 ± 1.0	< 0.01
UA, μmol/L	389.9 ± 89.3	419.1 ± 94.6	439.5 ± 97.3	446.2 ± 102.9	< 0.01
FPG, mmol/L	5.4 ± 0.6	5.6 ± 0.8	6.0 ± 1.7	7.4 ± 5.2	< 0.01
CAP, dB/m	313.3 ± 25.9	322.8 ± 29.3	332.6 ± 30.0	332.5 ± 34.1	< 0.01
LSM, Kpa	6.2 ± 4.1	6.8 ± 4.0	8.1 ± 5.2	8.5 ± 3.1	< 0.01

Data are presented as mean ± standard deviation (SD) for continuous variables or frequency (percentage) for categorical variables. *P*-values < 0.05 indicate statistical significance using ANOVA for continuous variables and chi-square tests for categorical variables.

Categorical variable definitions: (see Supplementary Table S7).

BMI, body mass index; WC, waist circumference; AC, abdominal circumference; HC, hip circumference; WHR, waist-to-hip ratio; ALT, alanine aminotransferase; AST, aspartate aminotransferase; ALP, alkaline phosphatase; GGT, gamma-glutamyl transferase; TG, triglycerides; TC, total cholesterol; HDL-C, high-density lipoprotein cholesterol; LDL-C, low-density lipoprotein cholesterol; FBG, fasting blood glucose; UA, uric acid; CAP, controlled attenuation parameter; LSM, liver stiffness measurement.

All *P*-values are two-sided with significance threshold α = 0.05.

Quartile cutoffs: Q1 (< 39.1), Q2 (39.1–43.6), Q3 (43.6–49.7), Q4 (≥49.7).

Sample size verification: 794 + 791 + 467 + 104 = 2,156 participants.

Missing data: None for variables included in this table.

### 3.2 Ethnic-specific threshold effects

**Figure 3:**
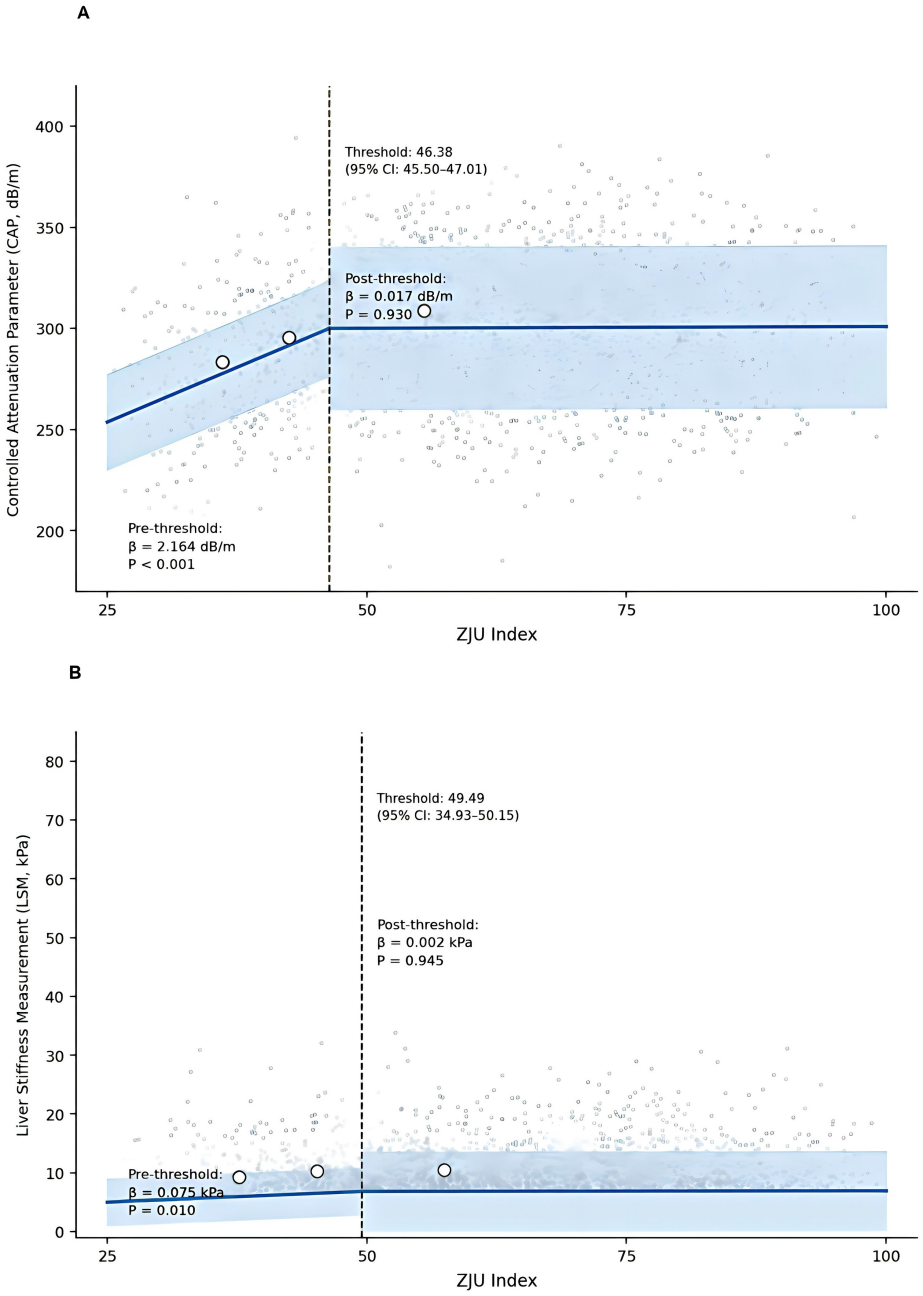
Simplified piecewise relationships between ZJU Index and VCTE Parameters Individual observations are shown in gray; fitted piecewise regression lines are shown in blue with 95% confidence bands. **(A)** ZJU index and hepatic steatosis (CAP). Piecewise regression model with patient-level data (*n* = 2,156). **(B)** ZJU index and liver fibrosis (LSM). Piecewise regression model with patient-level data (*n* = 2,156).

A restricted cubic spline analysis showed non-linear associations between the ZJU index and liver fat accumulation, and fibrous tissue formation (*P* < 0.001 for non-linearity). This indicated medically necessary population-tailored inflection points that were much lower compared to Western cohorts. Hepatic steatosis, ZJU index = 46.38 (95% CI: 45.50–49.44), was a critical inflection point; below it, each unit ZJU increase was associated with a steeper rise of 2.164 dB/m in CAP (95% CI: 1.734–2.595, *P* < 0.001), but after this threshold, associations became relatively flat (β = 0.017, *P* = 0.930). Likewise, liver fibrosis had a threshold at ZJU index = 49.49 (95% CI: 34.93–50.15), with a significant increase of 0.075 kPa per unit (95% CI: 0.018–0.133, *P* = 0.010) before this point and negligible beyond it (β = 0.002, *P* = 0.945). Both the piecewise models were found to be a better fit than the linear alternative (CAP: log-likelihood ratio *P* < 0.001; LSM: log-likelihood ratio *P* = 0.130), and bootstrap validation was used to confirm the stability of the thresholds (CAP: 0.005 optimism; LSM: 0.005 optimism; Table 2, Figure 3).

**Table 2:** Threshold effects of ZJU index on hepatic steatosis and fibrosis.

Parameter	Hepatic steatosis (CAP, dB/m)	Liver fibrosis (LSM, kPa)	*P*-value
Chinese MASLD cohort (*n* = 2,156)			
Inflection point (95% CI)	46.38 (45.50–49.44)	49.49 (34.93–50.15)	—
Pre-threshold slope β (95% CI)^†^	2.164 (1.734–2.595)	0.075 (0.018–0.133)	< 0.001/0.010
Post-threshold slope β (95% CI)^†^	0.017 (−0.367 to 0.401)	0.002 (−0.062 to 0.067)	0.930/0.945
Slope difference (*Δβ*)	2.147	0.073	< 0.001/0.035
Model fit (adjusted *R*^2^)	0.371	0.191	—
Bootstrap success rate	1,000/1,000 (100%)	100/100 (100%)	—
US NAFLD cohort-comparison (*n* = 2,122)^*^			
Inflection point	60.56	51.27	—
Pre-threshold slope β (95% CI)^†^	2.881 (2.625–3.137)	0.060 (0.024–0.096)	< 0.001
Post-threshold slope β (95% CI)	0.389 (−0.379 to 1.157)	0.296 (0.249–0.344)	NS/ < 0.001
Ethnic difference (Chinese vs. US)			
Threshold difference (absolute)	−14.18	−1.78	—
Threshold difference (relative %)	−23.4%	−3.5%	—
Pre-threshold slope difference	−0.717 (−24.9%)	+0.015 (+25.0%)	—
Post-threshold pattern	Plateau in both	Plateau (CN) vs. continued rise (US)	—
Quartile analysis (fully adjusted model 3)			
Q1 (< 39.1)-reference	—	—	—
Q2 (39.1–43.6)	+8.33 (5.25–11.40)	+0.03 (−0.42 to 0.49)	< 0.001/0.883
Q3 (43.6–49.7)	+17.29 (13.29–21.28)	+0.78 (0.19–1.36)	< 0.001/0.010
Q4 (≥49.7)	+17.19 (10.54–23.83)	+0.95 (−0.02 to 1.93)	< 0.001/0.056
*P* for trend	< 0.001	0.007	—

Piecewise linear regression with bootstrap validation in Chinese MASLD patients.

CAP, controlled attenuation parameter; CI, confidence interval; CN, Chinese; LSM, liver stiffness measurement; MASLD, metabolic dysfunction-associated steatotic liver disease; NS, not significant; US, United States; ZJU, Zhejiang University index.

Statistical methods: Piecewise linear regression with automatic inflection point detection using segmented regression. All models adjusted for age, sex, education, occupation, income level, waist circumference (WC), hip circumference (HC), waist-to-hip ratio (WHR), body fat percentage (BFP), laboratory parameters [alkaline phosphatase (ALP), gamma-glutamyl transferase (GGT), total cholesterol (TC), high-density lipoprotein cholesterol (HDL-C), low-density lipoprotein cholesterol (LDL-C), uric acid (UA)], dietary habits, taste preference, exercise frequency, daily routine (Model 3 specification).

^†^Bootstrap confidence intervals based on 1,000 iterations with bias-corrected percentile method.

LSM analysis used 100 bootstrap iterations for computational efficiency; validation confirmed threshold stability.

Critical divergence: US cohort demonstrates continued LSM increase post-threshold (β = 0.296, P < 0.001), indicating ongoing metabolic-driven fibrogenesis. Chinese cohort shows plateau (β = 0.002, P = 0.945), suggesting earlier transition to autonomous fibrosis phase independent of ZJU.

^*^US data from: Luo et al. (21). Correlation between ZJU index and hepatic steatosis and liver fibrosis in American adults with NAFLD.

Complete model specifications, alternative model comparisons (linear, restricted cubic spline), detailed bootstrap validation results, and sensitivity analyses are provided in Supplementary Tables S1–S6.

**Figure 4:**
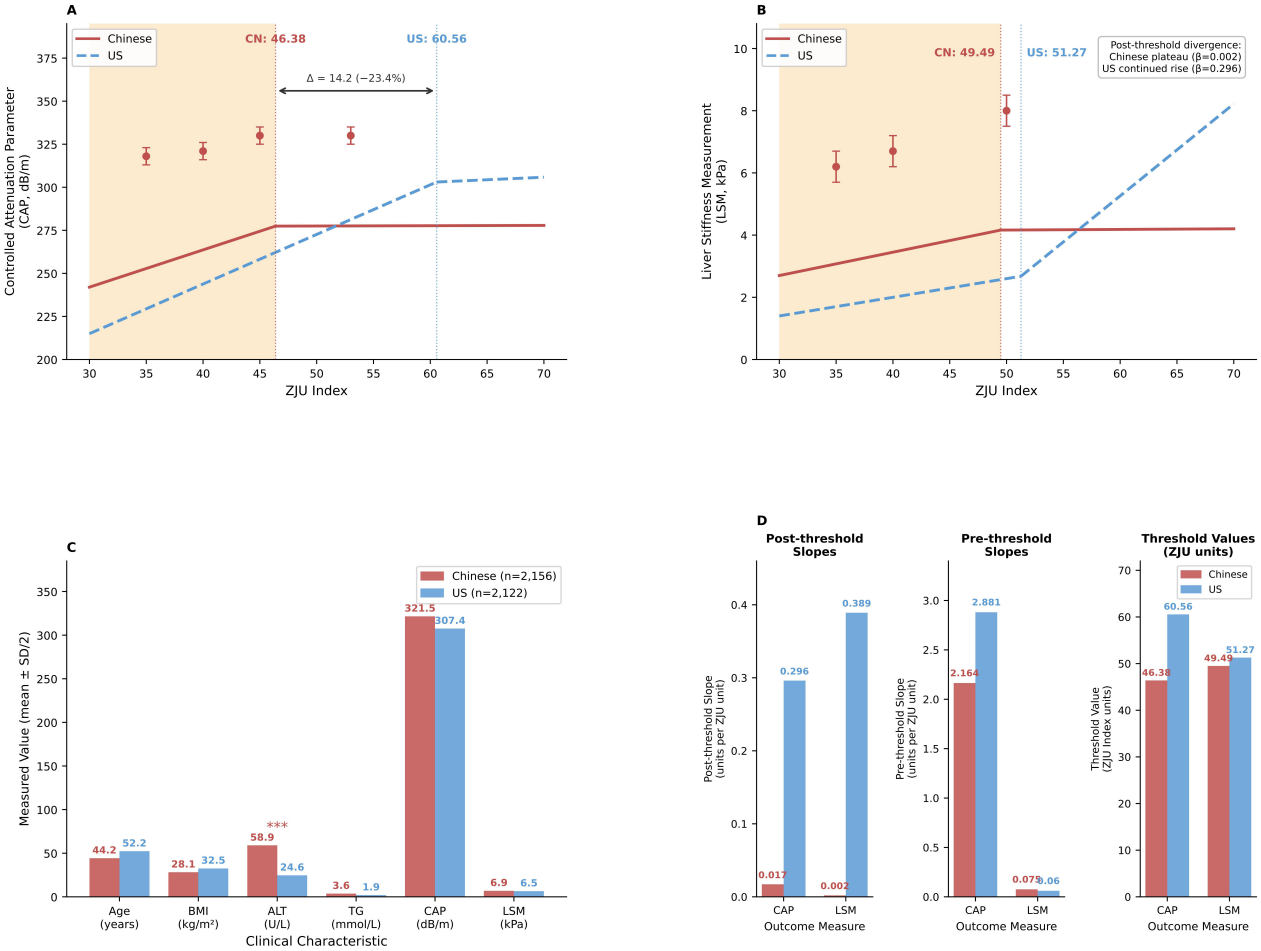
Ethnic-specific ZJU index thresholds: Chinese vs. US comparison. **(A)** Hepatic steatosis threshold comparison, **(B)** liver fibrosis threshold comparison, **(C)** key population characteristics, **(D)** threshold parameters summary. Data sources: Chinese cohort (*n* = 2,156, current study); US cohort [*n* = 2,122, Luo et al. (21)]. Solid lines = Chinese; dashed = US. Shaded regions indicate pre-threshold phases. ***P < 0.001 for Chinese vs. US difference. License: Creative Commons Attribution License (CC BY 4.0). Source: Frontiers in Medicine (open access).

These Chinese-specific inflection points were much lower than the previously established points for Western populations. For a more direct visualization of the critical finding that there are lower ethnic-specific thresholds, Figure 4 provides a comprehensive view of how the Chinese and US cohorts differ in terms of their threshold values, pre-threshold and post-threshold characteristics, and key demographics.

### 3.3 Linear associations and dose–response relationships

A multivariable linear regression analysis revealed robust positive associations between the ZJU index and VCTE parameters after full adjustment for demographic, metabolic, and lifestyle covariates. For every one-unit increase in the ZJU index, CAP and LSM increased by 0.96 dB/m (95% CI: 0.70–1.22, *P* < 0.001) and 0.042 kPa (95% CI: 0.004–0.080, *P* = 0.029), respectively (Figure 3). A quartile-stratified analysis revealed pronounced dose–response gradients. Compared to Q1, CAP elevations were Q2 = 8.33 dB/m (95% CI: 5.25–11.4), Q3 = 17.29 dB/m (95% CI: 13.29–21.28), and Q4 = 17.19 dB/m (95% CI: 10.54–23.83), with a significant trend *P* < 0.001. Progressive increases were observed in LSM: Q2 = 0.03 kPa (95% CI: −0.42 to 0.49, *P* = 0.883), Q3=0.78 kPa (95% CI: 0.19–1.36, *P* = 0.010), Q4 = 0.95 kPa (95% CI: −0.02 to 1.93, *P* = 0.056), with a significant trend *P* = 0.007 (Table 3).

**Table 3:** Linear regression analysis of ZJU index associations with VCTE parameters.

Variable	Model 1 (unadjusted)	Model 2 (demographics)	Model 3 (fully adjusted)^†^
Hepatic steatosis (CAP, dB/m)			
ZJU (continuous)			
β (95% CI)	1.180 (0.970–1.400)	1.170 (0.950–1.390)	0.960 (0.700–1.220)
*P*-value	< 0.001	< 0.001	< 0.001
ZJU (quartiles)			
Q1 (< 39.1)-reference	—	—	—
Q2 (39.1–43.6)	9.50 (6.69–12.31)	9.36 (6.54–12.18)	8.33 (5.25– 11.40)
Q3 (43.6–49.7)	19.29 (16.03–22.54)	19.16 (15.89–22.44)	17.29 (13.29–21.28)
Q4 (≥49.7)	19.23 (13.40–25.06)	18.95 (13.10–24.81)	17.19 (10.54–23.83)
*P* for trend	< 0.001	< 0.001	< 0.001
Liver fibrosis (LSM, kPa)			
ZJU (continuous, per unit)			
β (95% CI)	0.120 (0.090–0.150)	0.120 (0.080–0.150)	0.042 (0.004–0.080)
*P*-value	< 0.001	< 0.001	0.029
ZJU (quartiles)			
Q1 (< 39.1)-reference	—	—	—
Q2 (39.1–43.6)	0.59 (0.17–1.02)	0.58 (0.15–1.00)	0.03 (−0.42 to 0.49)
Q3 (43.6–49.7)	1.89 (1.40–2.38)	1.87 (1.38–2.36)	0.78 (0.19–1.36)
Q4 (≥49.7)	2.28 (1.41–3.16)	2.23 (1.35–3.11)	0.95 (−0.02 to 1.93)
*P* for trend	< 0.001	< 0.001	0.007

Progressive covariate adjustment in Chinese MASLD patients (n = 2,156).

CAP, controlled attenuation parameter; CI, confidence interval; LSM, liver stiffness measurement; MASLD, metabolic dysfunction-associated steatotic liver disease; VCTE, vibration-controlled transient elastography; ZJU, Zhejiang University index.

Model 1 (unadjusted): ZJU index only, no covariates.

Model 2 (demographics): Adjusted for age, sex, and occupational category.

Model 3 (fully adjusted): adjusted for age, sex, education, occupation, income level, waist circumference (WC), hip circumference (HC), waist-to-hip ratio (WHR), body fat percentage (BFP), laboratory parameters (alkaline phosphatase (ALP), gamma-glutamyl transferase (GGT), total cholesterol (TC), high-density lipoprotein cholesterol (HDL-C), low-density lipoprotein cholesterol (LDL-C), uric acid (UA), dietary habits, taste preference, exercise frequency, daily routine.

^†^Unless otherwise specified, β coefficients refer to Model 3 (fully adjusted). SD for ZJU index = 5.66 units.

β coefficients represent change in CAP (dB/m) or LSM (kPa) per unit increase in ZJU index or compared to eference quartile (Q1). Quartile analyses provide dose-response assessment. P-values < 0.05 indicate statistical significance.

### 3.4 Effect modification by clinical subgroups

**Figure 5:**
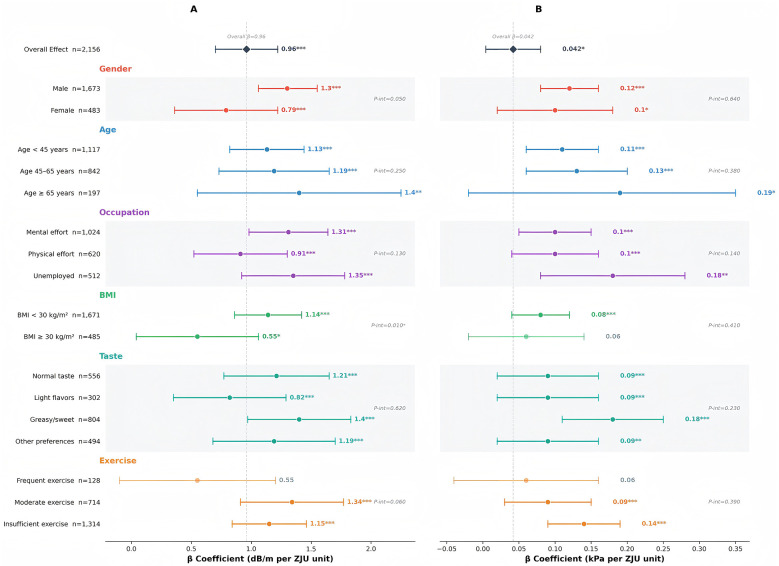
Subgroup analysis: ZJU index associations with VCTE parameters. **(A)** CAP (hepatic steatosis), **(B)** LSM (liver fibrosis). All estimates from fully adjusted model 3. *P*-int: *P*-value for interaction term testing effect modification. *P < 0.05, **P < 0.01, ***P < 0.001. All tests two-sided with α = 0.05 (interactions: α = 0.10).

Stratified analyses revealed significant heterogeneity in ZJU index-VCTE relationships across key clinical subgroups. Males exhibited stronger associations with hepatic steatosis than females (β = 1.30, 95% CI: 1.06–1.55 vs. β = 0.79, 95% CI: 0.36–1.22; *P*-interaction = 0.050), while elderly patients (≥65 years) showed the most pronounced correlations (β = 1.40, 95% CI: 0.55–2.25). The most clinically significant interaction occurred with obesity status: non-obese patients (BMI < 30 kg/m^2^) maintained robust ZJU index-CAP associations (β = 1.14, 95% CI: 0.86–1.42), whereas obese individuals showed markedly attenuated relationships (β = 0.55, 95% CI: 0.04–1.06; *P*-interaction = 0.010). Similar patterns were observed in liver fibrosis, with obesity completely eliminating ZJU index-LSM associations in patients with a BMI of ≥30 kg/m^2^ (β = 0.06, *P* = 0.112) while remaining significant in non-obese individuals (β = 0.08, *P* < 0.001), suggesting that profound adiposity-driven systemic inflammation may override the milder metabolic variations captured by the ZJU index. Exercise patterns revealed divergent effects: frequent exercisers showed non-significant CAP associations (β = 0.55, *P* = 0.096), contrasting with strong correlations in moderate exercisers (β = 1.34, *P* < 0.001; Table 4, Figure 5).

**Table 4:** Subgroup analysis of ZJU index associations with hepatic steatosis and fibrosis.

Subgroup	*n*	Hepatic steatosis (CAP)		Liver fibrosis (LSM)		*P* for interaction
		β (95% CI)	*P*-value	β (95% CI)	*P*-value	
Stratified by gender						
Male	1,673	1.30 (1.06–1.55)	< 0.001	0.12 (0.09–0.16)	< 0.001	0.050 (CAP) 0.640 (LSM)
Female	483	0.79 (0.36–1.22)	< 0.001	0.10 (0.02–0.19)	0.014	
Stratified by age						
< 45 years	1,117	1.13 (0.84–1.43)	< 0.001	0.11 (0.06–0.15)	< 0.001	0.250 (CAP) 0.380 (LSM)
45–65 years	842	1.19 (0.85–1.53)	< 0.001	0.13 (0.08–0.18)	< 0.001	
≥65 years	197	1.40 (0.55–2.25)	0.001	0.19 (0.03–0.35)	0.019	
Stratified by occupation						
Mental effort	1,024	1.31 (1.01–1.62)	< 0.001	0.10 (0.06–0.13)	< 0.001	0.130 (CAP) 0.140 (LSM)
Physical effort	620	0.91 (0.51–1.31)	< 0.001	0.10 (0.06–0.15)	< 0.001	
Unemployed	512	1.35 (0.88–1.82)	< 0.001	0.18 (0.07–0.29)	0.001	
Stratified by BMI						
< 30 kg/m^2^	1,671	1.14 (0.86–1.42)	< 0.001	0.08 (0.04–0.12)	< 0.001	0.010^*^ (CAP)
≥30 kg/m^2^ (obese)	485	0.55 (0.04–1.06)	0.035	0.06 (−0.02 to 0.14)	0.112	0.410 (LSM)
Stratified by taste preference						
Normal taste	556	1.21 (0.81–1.60)	< 0.001	0.09 (0.03–0.14)	< 0.001	0.620 (CAP)
Light flavors	302	0.82 (0.33–1.30)	< 0.001	0.09 (0.04–0.13)	< 0.001	0.230 (LSM)
Greasy/sweet flavors	804	1.40 (1.01–1.80)	< 0.001	0.18 (0.11–0.25)	< 0.001	
Other preferences	494	1.19 (0.70–1.68)	< 0.001	0.09 (0.02–0.15)	0.006	
Stratified by exercise frequency						
Frequent	128	0.55 (−0.10 to 1.19)	0.096	0.06 (−0.04 to 0.16)	0.247	0.060 (CAP) 0.390 (LSM)
Moderate	714	1.34 (0.97–1.70)	< 0.001	0.09 (0.04–0.14)	< 0.001	
Insufficient	1,314	1.15(0.86–1.44)	< 0.001	0.14(0.10–0.19)	< 0.001	

Fully adjusted models stratified by demographic anthropometric, and lifestyle factors (n = 2,156).

BMI, body mass index; CAP, controlled attenuation parameter; CI, confidence interval; LSM, liver stiffness measurement; ZJU, Zhejiang University index.

Model adjustments: All models adjusted for age, sex, education, occupation, income level, waist circumference (WC), hip circumference (HC), waist-to-hip ratio (WHR), body fat percentage (BFP), laboratory parameters (alkaline phosphatase (ALP), gamma-glutamyl transferase (GGT), total cholesterol (TC), high-density lipoprotein cholesterol (HDL-C), low-density lipoprotein cholesterol (LDL-C), uric acid (UA), dietary habits, taste preference, exercise frequency, daily routine. In stratified analyses, the stratification variable was excluded from adjustment.

β coefficients: represent change in CAP (dB/m) or LSM (kPa) per unit increase in ZJU index within each subgroup. All β values are from fully adjusted linear regression models (Model 3 specification).

Sample size distribution: explicit sample sizes (n=) provided for each subgroup to facilitate interpretation and meta-analysis. Total n = 2,156 across all analyses. Some subgroups have smaller sample sizes, resulting in wider confidence intervals.

P for interaction: tests whether the association between ZJU and outcome differs significantly across subgroups using multiplicative interaction terms. ^*^P < 0.05 indicates significant effect modification.

### 3.5 Sensitivity analyses

Sensitivity analyses supported the robustness of the main findings. The results from multiple imputation were materially consistent with those from the complete-case analysis. For CAP, the regression coefficient for the ZJU index changed only slightly from 1.107 (95% CI: 0.890–1.324) in the complete-case analysis to 1.060 (95% CI: 0.823–1.298) after imputation. For LSM, the corresponding coefficient changed from 0.117 (95% CI: 0.084–0.149) to 0.107 (95% CI: 0.078–0.135). The multiple imputation results are presented in Supplementary Table S8, and the adjusted linear reference models are shown in Supplementary Table S9.

### 3.6 Comparative performance of the ZJU index

To further evaluate the clinical utility of the ZJU index, we compared its performance with conventional anthropometric and metabolic indicators. For fibrosis-related outcomes, the ZJU index showed moderate discriminatory ability and outperformed several metabolic indicators, including triglycerides, the ALT/AST ratio, and the TyG index; however, BMI and waist circumference showed slightly higher AUCs in some fibrosis models. For CAP-defined steatosis severity, all tested indicators showed only modest discrimination, and the ZJU index demonstrated comparable rather than clearly superior performance. The ROC comparison is shown in Supplementary Figure S3, and the detailed pairwise DeLong test results are presented in Supplementary Table S10.

## 4 Discussion

### 4.1 Validation of the ZJU index as a hepatic steatosis and fibrosis biomarker

This cross-sectional analysis of 2,156 Chinese patients with MASLD confirms the ZJU index, as well as the association between hepatic steatosis (CAP: β = 0.96 dB/m per unit, *P* < 0.001) and fibrosis (LSM: β = 0.042 kPa per unit, *P* = 0.029) after adjustment for 19 demographic, anthropometric, metabolic, and lifestyle covariates. These results extend prior validation studies demonstrating ZJU index superiority over competing metabolic indices. The main associations remained materially unchanged in sensitivity analyses, including multiple imputation, supporting the robustness of the observed relationship between the ZJU index and VCTE-derived liver outcomes.

Specifically, Wang et al. (19) first demonstrated that the ZJU index [area under the receiver operating characteristic curve (AUROC) = 0.821] outperformed the fatty liver index (FLI, AUROC = 0.731), hepatic steatosis index (HSI, AUROC = 0.764), LAP (AUROC = 0.763), and visceral adiposity index (VAI, AUROC = 0.701) for MASLD detection in Chinese populations. Fu et al. (43) further established the ZJU index as a powerful surrogate marker for liver fat content, showing strong correlations with imaging-confirmed hepatic steatosis (*r* = 0.742, *P* < 0.001) that accounted for 50.4% of CAP variance, substantially better than traditional markers including FLI (*r* = 0.681), HSI (*r* = 0.629), and BMI (*r* = 0.512). Luo et al. extended this validation to US populations, establishing ZJU index-VCTE correlations with a CAP threshold at 60.56 and an LSM threshold at 51.27 for disease severity stratification (19–21). The dose–response relationships in our quartile analyses (CAP trend, *P* < 0.001; LSM trend, *P* = 0.007) provide compelling evidence that the ZJU index is not merely a diagnostic tool but a quantitative biomarker of disease burden. The steep slope from Q1 to Q4 (CAP: +19. 2 dB/m, LSM: +2.3 kPa) shows ZJU index ability to discriminate among the mild, moderate, and severe disease stages, which is a crucial feature for clinical risk stratification that many other competing metabolic indices lack (19–21).

### 4.2 Ethnic-specific thresholds: pathophysiological and genetic basis

A significant discovery from this study is the substantially lower ZJU index turning point for hepatic steatosis (CAP) in our Chinese population (46.38) compared to the US population (CAP: 60.56) (21), a 14.2-unit (23.4%) lower threshold. This disparity is due to fundamental ethnic differences in metabolic phenotypes, rather than methodological artifacts, as supported by three lines of evidence.

First, East Asians tend to gain fat in the abdomen at lower body weights than Europeans, a phenomenon known as the “Asian metabolic phenotype” (25, 26, 31–33). Equivalent BMI (e.g., 28 kg/m^2^) has 48% more computed tomography (CT)-estimated visceral adipose tissue (*P* < 0.001) (33), a higher hepatic fat fraction [magnetic resonance imaging (MRI): 12.3 vs. 8.7% in Europeans, *P* < 0.01] (25), and a lower subcutaneous-to-visceral fat ratio (0.82 vs. 1.24, *P* < 0.001) (33). This preferential accumulation of visceral fat is driven by the “thrifty genotype” hypothesis, which proposes that populations historically exposed to food scarcity have evolved metabolic responses that favor energy storage (31, 32). However, in modern obesogenic environments, these adaptations predispose individuals to ectopic fat deposition and insulin resistance at lower levels of adiposity. Our data support this: Chinese Q4 patients (ZJU index ≥49.7) have very severe hepatic steatosis (CAP: 332.5 ± 34.1 dB/m^2^) with a mean BMI of 33.4 ± 5.4 kg/m^2^, similar to the mean BMI of 35–40 kg/m^2^ for US MASLD patients (21).

Second, the patatin-like phospholipase domain-containing protein 3 (PNPLA3) rs738409 C>G polymorphism has the most robust genetic effect on MASLD susceptibility worldwide, with notable ethnic differences in allele frequency and effect size (22, 24, 34). East Asians have a higher G allele frequency (~45%−50%) (24) than Europeans (~25%−30%). The effect sizes for MASLD are stronger in Asians (OR = 1.92, 95% CI: 1.54–2.39) than in Europeans (OR = 1.73, 95% CI: 1.54–1.95). The effect sizes for advanced fibrosis and cirrhosis are even stronger in Asians (OR = 1.86, 95% CI: 1.64–2.12). Mechanistically, the I148M variant (G allele) leads to a loss of PNPLA3 lipase activity and impaired mobilization of triglycerides from lipid droplets (22), and increased hepatic fat accumulation (11%−38% more liver fat per G allele) (22, 24). Our lower threshold (46.38) corresponds to a higher population-level genetic risk in Chinese cohorts, assuming a 45% G allele frequency and an odds ratio (OR) of 1.92. Approximately 70% of Chinese MASLD patients would carry at least one risk allele, compared to 50% of Europeans (24). Owing to this genetic load, our steatosis progresses more aggressively the lower our ZJU index is, so let us not wait.

Third, East Asians exhibit disproportionate insulin resistance compared to their BMI (25, 33), which drives increased hepatic *de novo* lipogenesis (DNL), the primary source of liver triglycerides in MASLD (35, 36). Smith et al. (35) showed that MASLD patients had a 3-fold higher DNL rate than controls (26% ± 3% vs. 8% ± 2%, *P* < 0.001), which is directly associated with fasting insulin levels (*r* = 0.71, *P* < 0.001). Roumans et al. (36) also showed that hepatic saturated fatty acid (SFA) content (a DNL marker) was inversely associated with hepatic insulin sensitivity (*r* = −0.68, *P* < 0.001). Our data support this: the pre-threshold ZJU index-CAP slope of the Chinese cohort (β = 2.164 dB/m) was 25% less than that of the US cohort (β = 2.881 dB/m) (21), but the threshold occurred 23.4% sooner (46.38 vs. 60.56). That is, Chinese patients accumulate hepatic fat at a slower per-ZJU index rate but attain critical steatosis levels at lower metabolic burdens—indicative of elevated baseline DNL activity.

Comparative analyses further showed that the ZJU index was not uniformly superior to all conventional indicators. In particular, simple anthropometric measures such as BMI and waist circumference yielded slightly higher AUCs for some fibrosis-related outcomes, whereas the ZJU index outperformed several metabolic markers. These findings suggest that the ZJU index may offer value as a composite metabolic indicator, although its incremental benefit over simple anthropometric measures may be context-dependent.

Collectively, these findings underscore the critical need for ethnicity-specific reference thresholds in MASLD screening. The confluence of the “Asian metabolic phenotype,” high prevalence of the PNPLA3 risk allele, and exaggerated insulin resistance-driven DNL creates a pathophysiological landscape where Chinese individuals develop clinically significant hepatic steatosis at substantially lower ZJU index values compared to Western populations. Using a universal, Western-derived threshold would lead to a significant underdiagnosis of MASLD in China, potentially delaying crucial lifestyle interventions or medical management. Therefore, our proposed lower threshold not only reflects the unique genetic and metabolic vulnerabilities of the Chinese population but also provides a more accurate and clinically relevant tool for the early detection and stratification of MASLD in this high-risk group.

### 4.3 Non-linear dynamics: mechanistic transitions in MASLD

The threshold-dependent relationship regarding hepatic steatosis (inflection point: ZJU index = 46.38) reveals distinct pathophysiological transitions during MASLD progression. The steep pre-threshold rise (β = 2.16 4dB/m, *P* < 0.001) indicates metabolic-driven lipid accumulation mediated primarily through insulin resistance-driven *de novo* lipogenesis (DNL) and impaired VLDL secretion (1, 37). Insulin resistance drives hepatic DNL in recent studies, with DNL contributing up to 38% of liver TGs in MASLD patients, directly correlated with plasma glucose and insulin (35). However, after the inflection point (ZJU index > 46.38), the CAP does not show a significant change (β = 0.017, *P* = 0.930), indicating a shift from metabolic-driven steatosis to inflammation-dominated pathophysiology. Impaired mitophagy promotes NOD-like receptor family pyrin domain containing 3 (NLRP3) inflammasome activation as MASLD progresses to MASH, with saturated fatty acids driving mitochondrial dysfunction and IL-1β production (38–40), a transition from simple steatosis to steatohepatitis.

For liver fibrosis, the ZJU index = 49.49; the biphasic relationship represents different phases of fibrogenesis. A significant pre-threshold association (β = 0.075 kPa, *P* = 0.010) indicates the early metabolic-driven activation of stellate cells. Transforming growth factor-β (TGF-β) is a master profibrogenic cytokine, and platelet-derived growth factor (PDGF) is the central proliferative mediator in hepatic stellate cells (HSCs) (41, 42). An elevated ALT/AST ratio (ZJU index component) indicates hepatocyte injury releases profibrogenic factors (e.g., PDGF and TGF-β) that activate HSCs via the phosphoinositide 3-kinase/protein kinase B (PI3K/Akt) and Janus kinase/signal transducer and activator of transcription (JAK/STAT) pathways (10, 12, 41). Beyond the inflection point (ZJU index > 49.49), the association between the ZJU index and liver stiffness measurement plateaued (β = 0.002, *P* = 0.945), indicating no further significant increase. At this stage, fibrogenesis may become partially autonomous, with activated HSCs forming autocrine loops through sustained TGF-β/Smad and PDGF signaling (10), leading to extracellular matrix (ECM) deposition independent of metabolic drive. This mechanistic decoupling clinically explains why severe obesity (BMI ≥ 30 kg/m^2^) eliminated the ZJU index-LSM association. In patients with profound obesity, massive visceral adiposity triggers severe, persistent systemic inflammation and autonomous fibrogenesis that overshadow the milder metabolic fluctuations quantified by the ZJU index. In contrast, non-obese patients retained a significant association (β = 0.08, *P* < 0.001), indicating that their early fibrotic progression remains highly dependent on the metabolic risk factors effectively captured by the ZJU index. In contrast, advanced fibrosis becomes independent of ZJU index-captured metabolic factors.

In summary, the non-linear ZJU index-CAP and ZJU index-LSM relationships delineate critical pathophysiological checkpoints in MASLD progression. The plateaus observed above ZJU index 46.38 (steatosis and fibrosis) are not mere statistical artifacts but likely reflect fundamental mechanistic transitions: from reversible, metabolism-driven lipid accumulation to irreversible, inflammation-mediated hepatocellular injury, and from paracrine fibrogenesis to autonomous scar formation. These inflection points underscore the ZJU index's unique ability to act as a “molecular switch” detector, potentially guiding clinicians to intervene with metabolic therapies [e.g., lifestyle modification, glucagon-like peptide-1 (GLP-1) agonists] during the dynamic pre-threshold phase before the onset of self-sustaining inflammatory and fibrogenic cascades. Mechanistically, the individual components of the ZJU index directly mirror this DNL-driven pathogenesis. Elevated fasting blood glucose (FBG) reflects hepatic insulin resistance, which paradoxically upregulates SREBP-1c (sterol regulatory element-binding protein-1c) to hyperactivate DNL, resulting in the overproduction and systemic release of triglycerides (TGs). Concurrently, an increased ALT/AST ratio serves as an early, sensitive surrogate for the hepatocellular stress and lipotoxicity induced by this rapid intrahepatic lipid accumulation.

### 4.4 Clinical implications and ZJU index as a non-invasive screening tool

Our results demonstrate the feasibility of the ZJU index as a viable alternative to liver biopsy and VCTE for risk stratification of MASLD in Chinese individuals. ZJU index threshold (CAP: 46.38; LSM: 49.49) allows a three-tier risk stratification of low risk (ZJU index < 46.38), minimal/mild steatosis with lifestyle changes, moderate risk (ZJU index: 46.38–49.49), significant steatosis ± early fibrosis with consideration for pharmacotherapy, and high risk (ZJU index ≥49.49), advanced steatosis/fibrosis with specialty referral. This stratification accuracy mirrors VCTE performance; we found CAP increases of 8.33–17.29–17.19 dB/m (Q2 → Q3 → Q4) in our quartile analysis, corresponding to the progression from mild to moderate to severe steatosis according to the established CAP cutoffs (27).

The ZJU index requires only simple measurements (total cost ~$25) compared to a liver biopsy costing $2,000–$5,000 and having a 0.3%−0.5% complication rate (16) or VCTE, which costs between $50 and $300 per exam and over $50,000 for the equipment (17, 18). In rural China, where resource-limited areas are seeing a fast rise in MASLD and access to advanced imaging is limited, the ZJU index serves as an accessible first-line screening tool, with VCTE/biopsy reserved for high-risk cases (ZJU ≥ 49.49), and the strategy potentially cutting down on diagnosis cost by 60%−80% without affecting sensitivity (19–21). Unlike biopsy, serial biopsies are not practical or ethical, and unlike VCTE, patients have to travel. The ZJU index allows for frequent monitoring in the primary care setting. Our dose–response data suggest that ZJU index changes of ≥5 units (~1 SD) are associated with significant clinical steatosis/fibrosis shifts (CAP: ~5 dB/m change, LSM: ~0.2 kPa change), making the ZJU index suitable for response monitoring.

Taken together, these findings support the ZJU index as a practical, cost-effective, and accessible non-invasive tool for MASLD screening and risk stratification in Chinese populations. Its ability to identify distinct disease stages, facilitate early intervention, and enable serial monitoring in resource-limited settings addresses key limitations of both liver biopsy and VCTE. By providing a clear three-tier risk classification, the ZJU index may help standardize MASLD assessment across diverse clinical environments, particularly in rural China, where the disease burden is growing rapidly. Ultimately, the ZJU index offers a pragmatic solution to improve the scalability and efficiency of MASLD care, supporting more targeted use of advanced diagnostics and therapeutic resources.

### 4.5 Limitations and future directions

This study has several limitations. First, the cross-sectional design precludes establishing a causal relationship between ZJU index changes and MASLD progression. Second, the ethnic-specific thresholds (CAP: 46.38; LSM: 49.49) were derived from a Southwest Chinese cohort and may lack generalizability to the broader Asian population due to regional variations in PNPLA3 genetic heterogeneity and dietary patterns. Third, we lacked liver biopsy confirmation; while VCTE served as the reference standard, it has inherent diagnostic limitations (CAP sensitivity/specificity for ≥S2 steatosis: 69%−87% and 82%−91%, respectively; LSM AUROC for ≥F2 fibrosis: 0.84–0.89 with 10%−15% misclassification).

Future research should address these limitations through prospective, multi-center longitudinal cohorts across diverse Chinese regions. Implementing serial ZJU index measurements in these populations will help evaluate its dynamic predictive value for MASLD progression and therapeutic response. Furthermore, stratifying participants by regional dietary patterns and PNPLA3 allele frequencies is needed to externally validate and recalibrate these ethnic-specific thresholds. Finally, to overcome the diagnostic limitations of VCTE, future studies should incorporate paired liver biopsies for patients in threshold “gray zones” or utilize multiparametric magnetic resonance imaging [MRI-PDFF (magnetic resonance imaging proton density fat fraction) and MRE (magnetic resonance elastography)] to robustly refine the index's accuracy for early-stage fibrogenesis.

### 4.6 Conclusion

In conclusion, we established ethnic-specific ZJU index thresholds (CAP: 46.38; LSM: 49.49) for assessing hepatic steatosis and fibrosis in Chinese MASLD patients, which are significantly lower than Western thresholds. The ZJU index serves as a highly accessible, cost-effective, and non-invasive tool for accurate disease risk stratification, particularly in resource-limited settings. By applying these population-tailored cutoffs, clinicians in primary care can reliably identify high-risk patients, guide early lifestyle or pharmacological interventions, and optimize the allocation of advanced diagnostic resources without relying on expensive imaging or invasive biopsies.

## Data Availability

The original contributions presented in the study are included in the article/Supplementary material, further inquiries can be directed to the corresponding author.
