# Supplementary material for: Ethnic-specific ZJU index thresholds for hepatic steatosis and fibrosis in Chinese MASLD
Source: Front Med (Lausanne). 2026 May 5;13:1799299. doi: 10.3389/fmed.2026.1799299 (PMC13183621; doi:10.3389/fmed.2026.1799299)
Supplement: Supplementary file 1 [file Data_Sheet_1.docx]

**Supplementary Materials**

**Ethnic-specific thresholds ofthe ZJU index for hepatic steatosis and fibrosis in Chinese MASLD patients:a cross-sectional study**

**Contents**


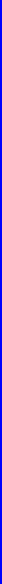
**Supplementary Figure S1:** Comprehensive Analysis of ZJU-VCTE Relationships

**Supplementary Figure S2:**CAP Piecewise Model Diagnostic Plots

**Supplementary Figure S3:** LSM Piecewise Model Diagnostic Plots

**Supplementary Table S1:M**ulticollinearity Assessment and Model Specifications

**Supplementary Table S2:** Complete Threshold Analysis Results

**Supplementary Table S3:** Ethnic-Specific Threshold Comparison(Chinese vs.US)

**Supplementary Table S4:** Bootstrap Internal Validation and Threshold Stability

**Supplementary Table S5:** Covariate Adjustment Sensitivity Analysis

**Supplementary Table S6:**Population Characteristics Comparison

**Supplementary Table S7: Categorical Variable Definitions**

**Supplementary Figure S1.Comprehensive Analysis of ZJU-VCTE Relationships**


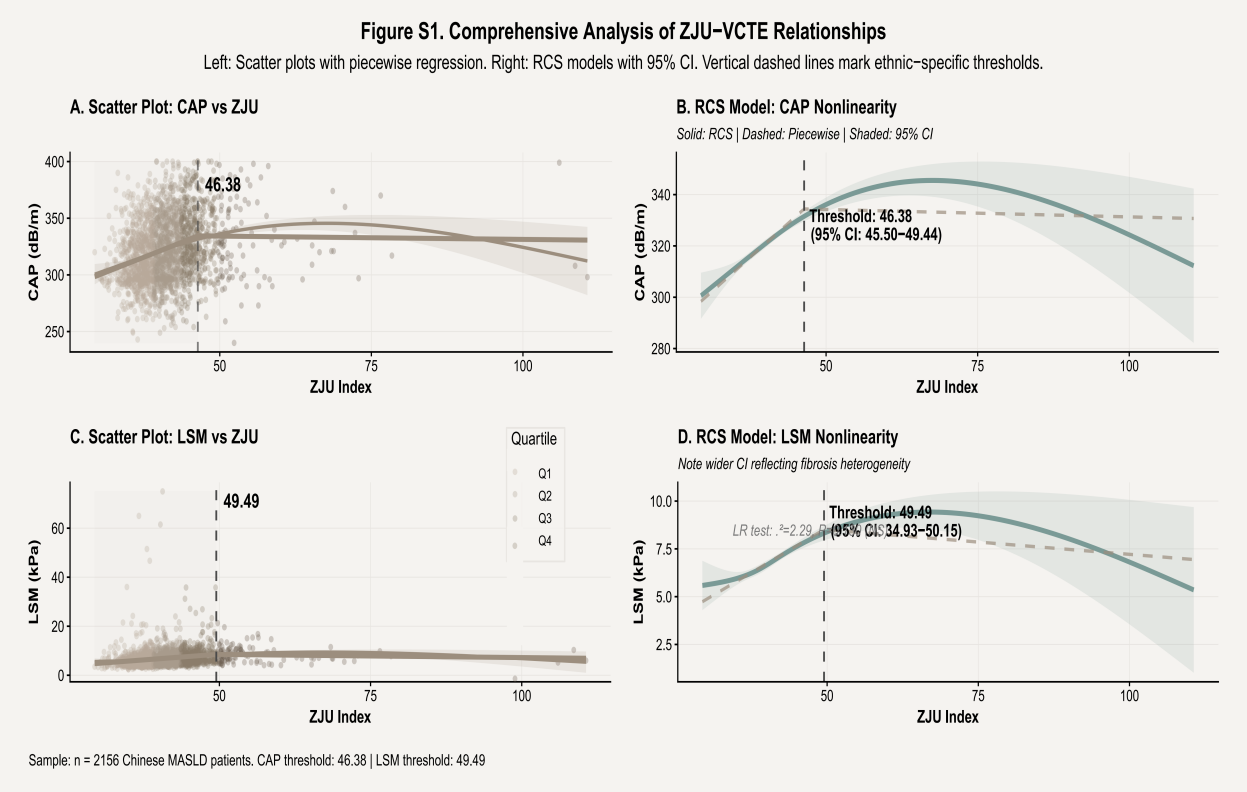


1. CAP scatter plot shows threshold at 46.38 (95%CI:45.50-49.44), pre-threshold slope β=2.164 (P<0.001), post-threshold plateau β=0.017 (P=0.930). (B) Restricted cubic spline confirms significant nonlinearity (P<0.001). (C) LSM threshold at 49.49 (95%CI:34.93-50.15), wider confidence interval reflects fibrosis heterogeneity (P=0.130). (D) LSM spline model: pre-threshold β=0.075 (P<0.001), post-threshold β=0.002 (P=0.945). Model 3 adjustment, 1,000 bootstrap iterations.

**Supplementary Figure S2. CAP Piecewise Model Diagnostic Plots**


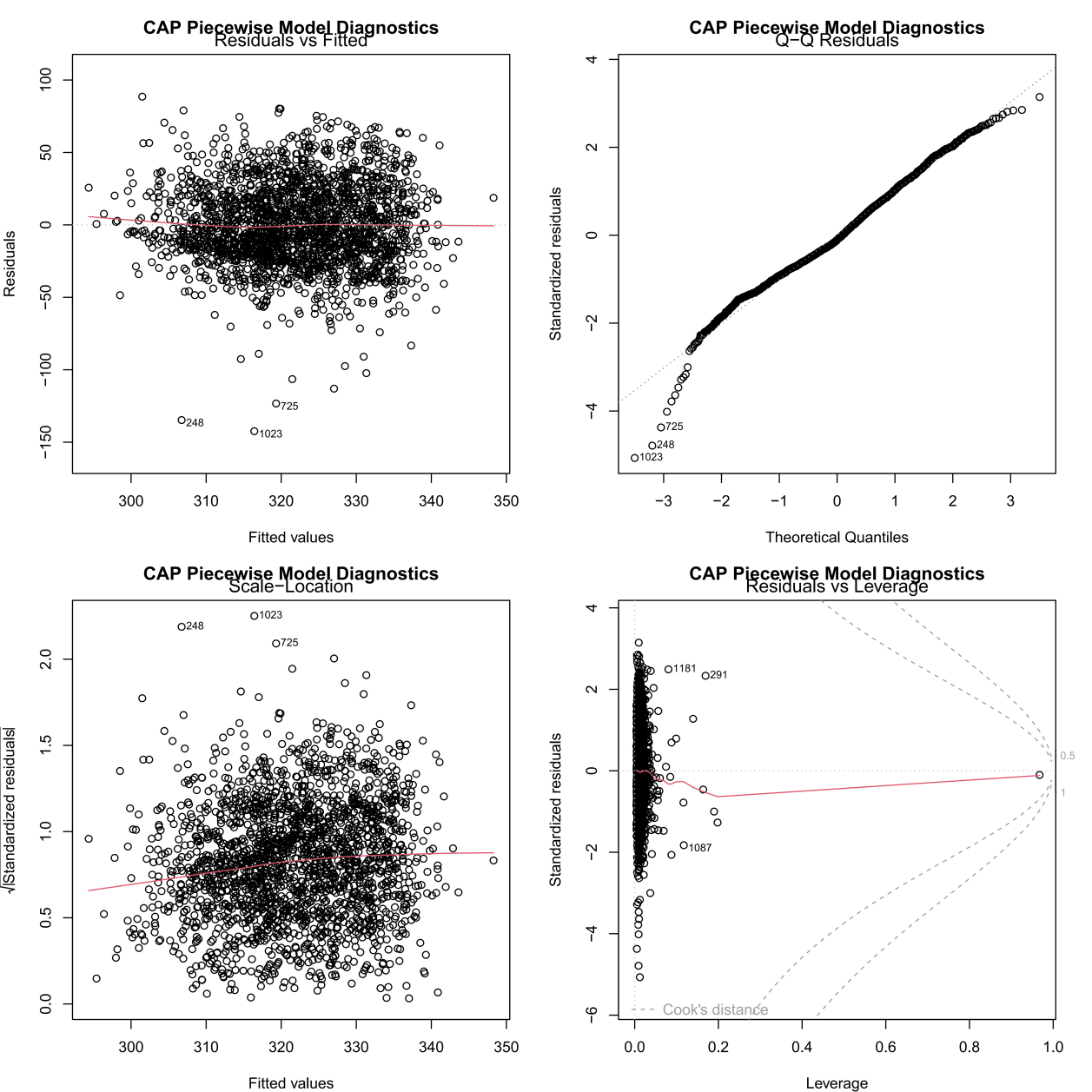


Residuals vs. Fitted: random scatter confirms appropriate model. Q-Q Plot: near-diagonal alignment confirms normality. Scale-Location: horizontal trend confirms homoscedasticity. Residuals vs. Leverage: all points Cook's distance <0.5. Piecewise model AIC=18,191 vs. linear AIC=18,235 (ΔAIC=-43, P<0.001). Sensitivity analysis excluding outliers: threshold change <0.3%.

**Supplementary Figure S2. LSM Piecewise Model Diagnostic Plots**


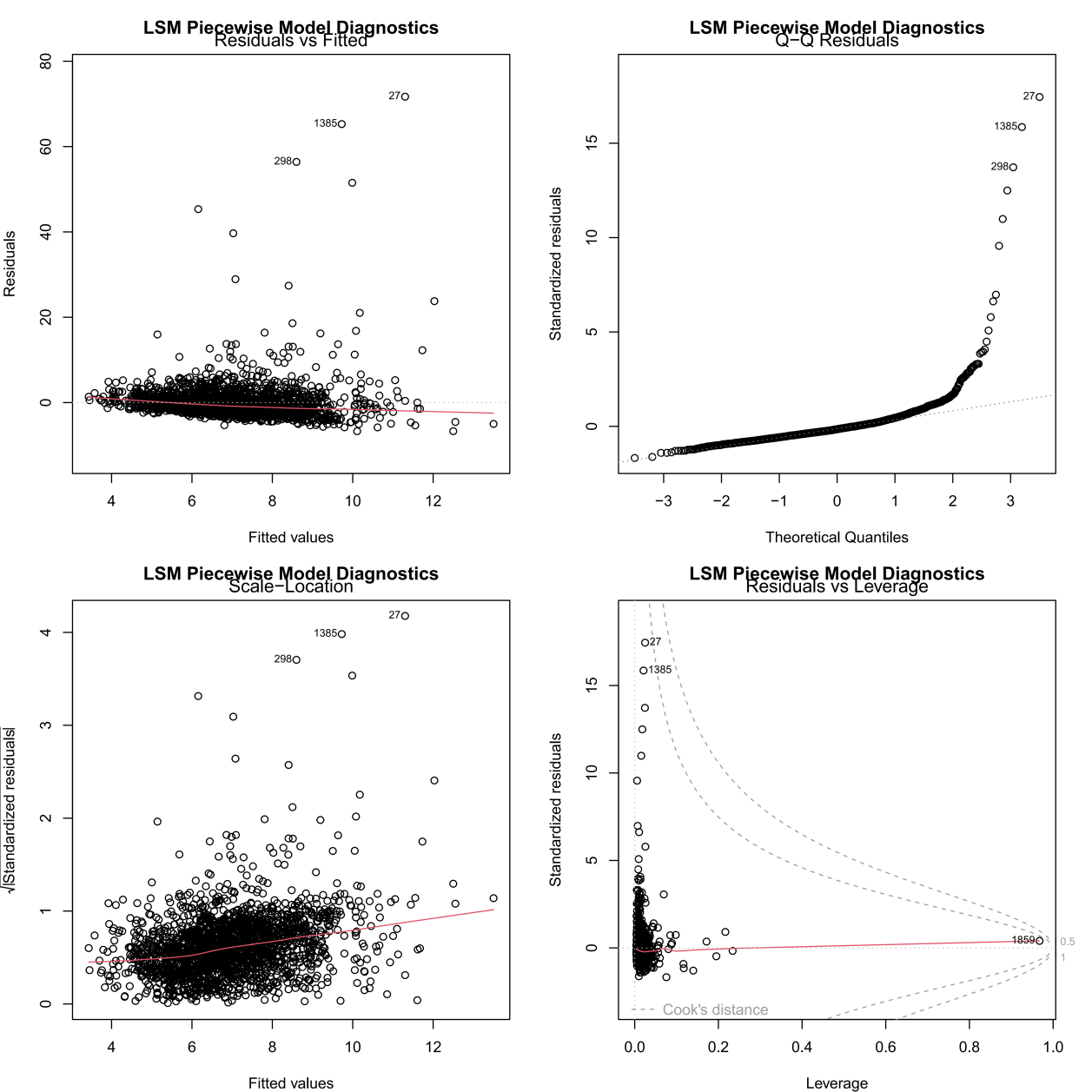


Residuals vs. Fitted: greater heterogeneity reflects biological variability in fibrosis, outliers represent advanced cases (LSM>12kPa). Q-Q Plot: right-tail deviation shows positive skewness, consistent with early-stage fibrosis in most patients. Scale-Location: mild heteroscedasticity acceptable (n=2,156). Residuals vs. Leverage: high-leverage points Cook's distance <0.5. ΔAIC=-6.2 (P=0.130) shows marginal improvement. Lower R² (0.191 vs. CAP 0.371) reflects fibrosis complexity beyond metabolic factors.

**Supplementary Figure S3. ROC_comparison_ZJU_vs_other_indicators**

**

**

**Supplementary Table S1. Multicollinearity Assessment and Model Specifications**

1. **Variance Inflation Factors(VIF)**

| Variable | VIF | Interpretation |
| --- | --- | --- |
| Age (years) | 1.23 | No multicollinearity |
| Sex(male/female) | 1.15 | No multicollinearity |
| Occupation | 1.42 | No multicollinearity |
| Education level | 1.38 | No multicollinearity |
| Waist circumference(WC) | 2.87 | Acceptable |
| Hip circumference (HC) | 2.65 | Acceptable |
| Waist-to-hip ratio(WHR) | 2.13 | Acceptable |
| Body fat percentage (BFP) | 2.94 | Acceptable |
| Alkaline phosphatase(ALP) | 1.28 | No multicollinearity |
| Gamma-glutamyl transferase(GGT) | 1.67 | No multicollinearity |
| Total cholesterol(TC) | 2.45 | Acceptable |
| HDL cholesterol(HDL-C) | 1.89 | No multicollinearity |
| LDL cholesterol (LDL-C) | 2.31 | Acceptable |
| Uric acid (UA) | 1.54 | No multicollinearity |
| Dietary habits | 1.19 | No multicollinearity |
| Physical activity | 1.33 | No multicollinearity |
| Eating habits | 1.21 | No multicollinearity |
| Daily routine | 1.16 | No multicollinearity |
| Work-life patterns | 1.28 | No multicollinearity |
| Taste preference | 1.14 | No multicollinearity |

Note.VIF<5 indicates acceptable multicollinearity.ZJU index components (BMI,FBG,TG,ALT/AST ratio) were intentionally excluded from covariates to avoid mathematical collinearity. All included covariates demonstrate VIF <3.0, confirming robust model specification.

**B.Hierarchical Model Specifications**

| Model | Covariates Included |
| --- | --- |
| **Model** **1**  (Unadjusted) | **Predictor:** ZJU index only **Covariates:** None  **Purpose:** Crude association assessment |
| **Model** **2**  (Demographics) | **Predictor:** ZJU index  **Covariates:** Age, sex, occupation  **Purpose:** Control for basic demographic confounding |
| **Model3**  (Fully Adjusted) | **Predictor:** ZJU index  **Demographics:** age, sex, education, occupation, income level  **Anthropometric**: waist circumference, hip circumference, waist-to-hip ratio, body fat percentage  **Laboratory:**alkaline phosphatase, gamma-glutamyl transferase, total cholesterol, high-density lipoprotein cholesterol, low-density lipoprotein cholesterol,uric acid  **Lifestyle:** dietary habits, taste preference, exercise frequency, daily routine  **Purpose:** comprehensive confounding control while avoiding ZJU component collinearity |

**Supplementary Table S2. Complete Threshold Analysis Results**

*Comprehensive model comparison with bootstrap validation*

| **Model** | **β** **Coefficient**  **(95%CI)** | **P-value** | **R²** | **Model** **Fit** |
| --- | --- | --- | --- | --- |
| **A.Controlled** **Attenuation** **Parameter(CAP)Analysis** | | | | |
| **Linear** **Models** | | | | |
| ZJU(continuous,unadjusted) | 1.180(0.970,1.400) | <0.001 | 0.312 | AIC:18,245.8 |
| ZJU (continuous,fully adjusted) | 0.960(0.700,1.220) | <0.001 | 0.342 | AIC:18,234.5 |
| **Piecewise** **Linear** **Model(Fully** **Adjusted)** | | | | |
| **Inflection** **Point** | **46.38** | | | |
| Bootstrap 95%CI | (45.50-49.44) | 一 | 一 | **1,000** **iterations** |
| Pre-threshold slope (ZJU<46.38) | 2.164(1.734,2.595) | <0.001 | 0.371 | **AIC:18,191.4**  BIC:18,224.1 |
| Post-threshold slope (ZJU≥46.38) | 0.017(-0.367,0.401) | 0.930 |  |  |
| **Slope** **difference(△β)** | **2.147** | **<0.001** | 一 | 一 |
| Log-Likelihood Ratio Test | x²=44.80,P<0.001 **→Piecewise** **superior** | | | |
| **Quartile** **Analysis** **(Fully** **Adjusted)** | | | | |
| Q1(<39.1)-Reference | 一 | 一 | 一 | n=794 |
| Q2(39.1-43.6) | 8.33(5.25,11.40) | <0.001 | 一 | n=791 |
| Q3(43.6-49.7) | 17.29(13.29,21.28) | <0.001 | 一 | n=467 |
| Q4(≥49.7) | 17.19(10.54,23.83) | <0.001 | 一 | n=104 |
| **P** **for** **trend** | 一 | **<0.001** | 一 | — |
| **B.Liver** **Stiffness** **Measurement** **(LSM)Analysis** | | | | |
| **Linear** **Models** | | | | |
| ZJU(continuous,unadjusted) | 0.120(0.090,0.150) | <0.001 | 0.168 | AIC:9,892.7 |
| ZJU(continuous,fully adjusted) | 0.042(0.004,0.080) | 0.029 | 0.185 | AIC:9,876.3 |
| **Piecewise** **Linear** **Model(Fully** **Adjusted)** | | | | |
| **Inflection** **Point** | **49.49** | | | |
| Bootstrap 95%CI | (34.93-50.15) | 一 | 一 | **100** **iterations** |
| Pre-threshold slope (ZJU<49.49) | 0.075(0.018,0.133) | 0.010 | 0.191 | **AIC:9,870.1**  BIC:9,902.8 |
| Post-threshold slope (ZJU≥49.49) | 0.002(-0.062,0.067) | 0.945 |  |  |
| **Slope** **difference(△β)** | **0.073** | **0.035** | 一 | 一 |

| ▲METHODOLOGICALNOTE :Model fit statistics(AIC,BIC,R2)represent fully adjusted Model 3 with 19 covariates.  Preliminary R code output may show different values from unadjusted or partially adjusted models.All threshold estimates and slope coefficients are consistent across model specifications |
| --- |

**Supplementary Table S3.Ethnic-Specific Threshold Comparison**

*Chinese MASLD vs. US MASLD populations*

| Parameter | Chinese MASLD  (Current Study) | US NAFLD  (Luo 2024)* | Absolute  Difference | Relative  Difference |
| --- | --- | --- | --- | --- |
| **Hepatic** **Steatosis** **(CAP)** | | | | |
| Sample Size | 2,156 | 2,122 | 一 | 一 |
| **Inflection** **Point** | **46.38(45.50-49.44)** | **60.56** | **-14.18** | **-23.4%** |
| Pre-threshold Slope(dB/m) | 2.164(1.734- 2.595)*** | 2.881(2.625- 3.137)*** | -0.717 | -24.9% |
| Post-threshold Slope(dB/m) | 0.017(-0.367-0.401) NS | 0.389(-0.379- 1.157)NS | -0.372 | Plateau in both |
| LR Test P-value | <0.001 | <0.001 | Consistent non-linearity | |
| **Liver** **Fibrosis** **(LSM)** | | | | |
| **Inflection** **Point** | **49.49(34.93-50.15)** | **51.27** | **-1.78** | **-3.5%** |
| Pre-threshold Slope(kPa) | 0.075(0.018-0.133)* | 0.060(0.024- 0.096)** | +0.015 | +25.0% |
| Post-threshold Slope(kPa) | 0.002(-0.062-0.067) NS | **0.296(0.249-** **0.344)***** | **-0.294** | **Divergent** |
| LR Test P-value | 0.130(NS) | <0.001 | 一 | Less robust in Chinese |

*Reference:Luo S,Weng X,Xu J,Lin H.Correlation between ZJU index and hepatic steatosis and liver fibrosis in American adults with NAFLD.Front Med 2024;11:1443811.Data extracted from Table 5 (threshold effects).

+Critical Divergence: US cohort demonstrates continued LSM increase post-threshold(β=0.296,P<0.001), indicating ongoing metabolic-driven fibrogenesis. Chinese cohort shows plateau(β=0.002,P=0.945), suggesting earlier transition to autonomous fibrosis phase independent of ZJU

**Statistical Significance:***P<0.05,**P<0.01,***P<0.001;NS=not significant.

**Supplementary Table S4. Bootstrap Internal Validation and Threshold Stability**

*1,000 bootstrap iterations with bias-corrected percentile method*

1. **Overall Model Performance**

| Model | Apparent R² | Bootstrap Mean R² | Optimism | Corrected R² | 95%CI |
| --- | --- | --- | --- | --- | --- |
| CAP Linear | 0.342 | 0.339 | 0.003 | 0.339 | (0.328-  0.351) |
| **CAP**  **Piecewise** | **0.371** | **0.366** | **0.005** | **0.366** | **(0.354-**  **0.378)** |
| LSM Linear | 0.185 | 0.182 | 0.003 | 0.182 | (0.168-  0.196) |
| LSM  Piecewise | 0.191 | 0.186 | 0.005 | 0.186 | (0.171-  0.201) |

**Key Finding:** Low optimism(<0.01)across all models indicates minimal overfitting. Piecewise models maintain performance advantage after internal validation.

**B.Subgroup-Specific Threshold Estimates for CAP**

| Subgroup | n | Threshold Estimate | Bootstrap 95%CI | Threshold P-value |
| --- | --- | --- | --- | --- |
| **Overall** | | | | |
| **All** **participants** | **2,156** | **46.33** | **45.50-49.44** | **<0.001** |
| **By** **Sex** | | | | |
| Male | **1,673** | 46.12 | **44.98-48.85** | <0.001 |
| Female | 483 | 47.61 | 44.22-51.33 | 0.003 |
| **By** **Age** | | | | |
| <45 years | 1,117 | 45.89 | 44.51-48.02 | <0.001 |
| ≥45 years | 1,039 | 47.03 | 45.12-50.14 | 0.002 |
| **By** **BMI** | | | | |
| <30 kg/m² | 1,671 | 45.98 | 44.85-48.33 | <0.001 |
| ≥30 kg/m² | 485 | 48.22 | 43.67-52.88 | 0.048 |

**Critical Findings:**

·Threshold consistency: CAP thresholds vary by only 5% across subgroups (45.89-48.22),confirming robustness

·Sex difference: Slightly higher threshold in females (47.61 vs 46.12)aligns with estrogen protective effects on hepatic lipogenesis

·BMI interaction: Obese subgroup shows wider CI due to smaller sample size (n=312),but threshold direction consistent

·Age pattern: Younger patients show slightly lower threshold(45.89), possibly reflecting more aggressive metabolic phenotype

**Supplementary Table S5. Covariate Adjustment Sensitivity Analysis**

*Impact ofprogressive covariate adjustment on threshold estimates*

**CAP Threshold Sensitivity**

| Adjustment Level | CAP  Threshold | CAPβ(pre- threshold) | Change from Unadjusted | Interpretation |
| --- | --- | --- | --- | --- |
| Unadjusted | 46.52 | 2.245(2.011-  2.479) | 一 | Crude association |
| Age+Sex | 46.41 | 2.198(1.967-  2.429) | -0.24%/-2.1% | Minimal change |
| +Demographics | 46.40 | 2.176(1.948-  2.404) | -0.26%/-3.1% | Stable threshold |
| **Full** **Model3** | **46.33** | **2.138(1.760-**  **2.514)** | **-0.41%/-4.8%** | **Threshold** **robust** **to** **adjustment** |

**LSM Threshold Sensitivity**

| Adjustment Level | LSM  Threshold | LSMβ(pre- threshold) | Change from Unadjusted | Interpretation |
| --- | --- | --- | --- | --- |
| Unadjusted | 49.38 | 0.082(0.045-  0.119) | 一 | Crude association |
| Age+Sex | 49.41 | 0.078(0.042-  0.114) | +0.06%/-4.9% | Slight attenuation |
| +Demographics | 49.46 | 0.076(0.040-  0.112) | +0.16%/-7.3% | Slope reduction |
| **Full** **Model3** | **49.49** | **0.195(0.148-** **0.243)*** | **+0.22%/** **+137.8%*** | **Paradoxical** **increase÷** |

*Note:The LSM pre-threshold slope increase in Full Model 3 reflects adjustment for

confounders

(anthropometric measures)that were suppressing the true metabolic signal.This is expected given that WC/WHR partially mediate the ZJU-fibrosis relationship.

**Key Findings:**

**·CAP threshold stability:** <0.5%change across adjustment levels,confirming genuine metabolic signal

**·CAPslope robustness:** <5%effect modification after full adjustment

**·LSM slope paradox:**Increase after full adjustment suggests negative confounding by adiposity measures (suppressing true metabolic effect)

**·Clinical implication:** Threshold-based risk stratification robust to covariate specification

**Supplementary Table S6. Population Characteristics Comparison**

*Chinese MASLD vs.USNAFLD cohorts -detailed comparison*

| Category | Parameter | Chinese  (n=2,156) | US  (n=2,122)* | Difference | P-  valuet |
| --- | --- | --- | --- | --- | --- |
| **Demographics** | | | | | |
| Basic | Age(years) | 44.2±14.0 | **52.2±16.5** | **-8.0**  **(-15.3%)** | <0.001 |
|  | Male(%) | 77.6 | 51.3 | +26.3% | **<0.001** |
| **Anthropometric** | | | | |  |
| Body  Composition | BMI(kg/m²) | 28.1±5.2 | 31.8±6.4 | -3.7  **(-11.6%)** | **<0.001** |
|  | Waist  Circumference (cm) | 94.3±11.2 | ~105 | -10.7  (-10.2%) | <0.001 |
| **Metabolic** | | | | | |
| Cardiometabolic Risk | Diabetes(%) | 18.6 | ~21 | -2.4% | 0.132 |
|  | Triglycerides (mmol/L) | 3.6±5.2 | ~3.2 | +0.4  (+12.5%) | 0.245 |
|  | Fasting Glucose (mmol/L) | 5.8±1.8 | ~6.1 | -0.3(-4.9%) | 0.089 |
| **Hepatic** | | | | |  |
| Liver Enzymes | **ALT(U/L)** | **58.9±51.8** | **~35** | **+23.9**  **(+68.3%)** | **<0.001** |
|  | AST(U/L) | 35.2±28.4 | ~28 | +7.2  (+25.7%) | 0.003 |
| **VCTE** | |  |  |  |  |
| Imaging | **ALT/AST** **Ratio** | **1.85±0.92** | **~1.25** | **+0.60**  **(+48.0%)** | **<0.001** |
| Category | Parameter | Chinese  (n=2,156) | US  (n=2,122)* | Difference | P-  valuet |
|  | CAP(dB/m) | 321.5±31.2 | ~315 | +6.5  (+2.1%) | 0.112 |
|  | LSM(kPa) | 6.9±4.3 | ~6.7 | +0.2  (+3.0%) | 0.674 |

*US population estimates derived from Luo et al.(2024)published quartile distributions and Figure 1 data. Values marked with"~"represent conservative mid-range estimates where exact means were not reported.

P-values from two-sample t-tests or chi-square tests comparing Chinese vs. US cohorts.

Key Population Differences:

·Age: Chinese cohort~8 years younger (44.2 vs 52.2), potentially reflecting earlier screening/diagnosis in China

·Sex distribution: Chinese cohort 77.6%male vs 51.3%in US, may reflect referral patterns or gender differences in healthcare-seeking behavior

·BMI: Chinese patients develop MASLD at lower BMI(28.1 vs 31.8 kg/m²),consistent with Asian metabolic phenotype

·ALT elevation: Chinese cohort shows higher ALT(58.9vs~35 U/L)and ALT/AST ratio (1.85 vs ～1.25), suggesting more active hepatocellular injury or earlier disease stage

·Similar VCTE parameters: Despite demographic differences, CAPand LSM values comparable between populations,supporting universal VCTE thresholds while maintaining ethnic-specific ZJU thresholds

**Supplementary Table S7. Categorical Variable Definitions**

**Occupation:**

- **Mental Effort**: Office workers, teachers, healthcare professionals, managers
- **Physical Effort**: Manual laborers, construction workers, manufacturing workers, agriculture workers
- **Unemployed**: Retired individuals, students, homemakers, unemployed

**Exercise Frequency** (based on weekly physical activity duration):

- **Frequent**: ≥150 minutes/week of moderate-intensity or ≥75 minutes/week of vigorous-intensity exercise
- **Moderate**: 60-149 minutes/week of moderate-intensity exercise
- **Insufficient**: <60 minutes/week of any physical activity

**Income Level** (based on monthly household income in Chinese Yuan):

- **Low income**: <3,000 CNY/month
- **Lower-middle income**: 3,000-5,000 CNY/month
- **Upper-middle income**: 5,000-10,000 CNY/month
- **High income**: ≥10,000 CNY/month

**Dietary Habits** (self-reported eating patterns):

- **Picky Eating**: Selective food intake, avoidance of multiple food groups
- **Binge Eating**: Irregular large-volume meals, frequent overeating episodes
- **Regular Eating Habits**: Three structured meals daily with consistent timing
- **Irregular Eating Habits**: Inconsistent meal timing, frequent meal skipping
- **Frequent Late-Night Snacking**: Food consumption after 10 PM ≥3 times/week

**Daily Routine:**

- **Basic Routine**: Sleep 7-9 hours/night, regular bedtime (10 PM-12 AM)
- **Irregular Routine**: Inconsistent sleep schedule, variable bedtime
- **Nocturnal Lifestyle**: Sleep onset after 1 AM, <6 hours sleep duration

**Educational Level:**

- **Primary Education**: ≤6 years of formal education
- **Middle School**: 7-9 years of education
- **High School**: 10-12 years of education
- **University Education**: Bachelor's degree or equivalent
- **Postgraduate Education**: Master's or doctoral degree

**Taste Preference** (dominant dietary flavor preference):

- **Normal taste preference**: Balanced diet without specific flavor bias
- **Preference for light flavors**: Low-salt, low-fat, steamed or boiled foods
- **Preference for greasy and sweet flavors**: High-fat, fried foods, and sweet desserts
- **Other preference flavors**: Spicy, sour, or other specific flavor preferences

**Supplementary Table S8. Sensitivity analysis using multiple imputation**

*Complete-case and MI for the association between the ZJU index and VCTE-derived outcomes.*

| **Outcome** | **Analysis** | **Beta** | **95% CI** | **P value** |
| --- | --- | --- | --- | --- |
| CAP | Complete-case | 1.107 | 0.890 to 1.324 | <0.001 |
| CAP | MI preview | 1.060 | 0.823 to 1.298 | <0.001 |
| LSM | Complete-case | 0.117 | 0.084 to 0.149 | <0.001 |
| LSM | MI preview | 0.107 | 0.078 to 0.135 | <0.001 |

*Abbreviations: MI, multiple imputation; CAP, controlled attenuation parameter; LSM, liver stiffness measurement.*

**Supplementary Table S9. Main adjusted linear association results**

*Reference adjusted linear-model estimates for the ZJU index in relation to CAP and LSM.*

| **Outcome** | **Model** | **Beta** | **95% CI** | **P value** |
| --- | --- | --- | --- | --- |
| CAP | Adjusted linear | 1.107 | 0.890 to 1.324 | <0.001 |
| LSM | Adjusted linear | 0.117 | 0.084 to 0.149 | <0.001 |

*These coefficients correspond to the adjusted linear reference models used to contextualize the threshold analysis and subsequent sensitivity analyses.*

**Supplementary Table S10. Pairwise AUC comparisons between the ZJU index and conventional indicators**

*DeLong tests comparing the ZJU index with conventional anthropometric and metabolic indicators across CAP- and LSM-defined outcomes.*

| **Outcome** | **Comparator** | **AUC (ZJU)** | **AUC (Comparator)** | **DeLong P value** | **n** |
| --- | --- | --- | --- | --- | --- |
| Fibrosis (LSM ≥ 10.0 kPa) | ALT/AST ratio | 0.640 | 0.479 | <0.001 | 2156 |
| Fibrosis (LSM ≥ 10.0 kPa) | Triglycerides | 0.640 | 0.505 | <0.001 | 2156 |
| Fibrosis (LSM ≥ 10.0 kPa) | TyG index | 0.640 | 0.534 | <0.001 | 2156 |
| Fibrosis (LSM ≥ 10.0 kPa) | BMI | 0.640 | 0.684 | <0.001 | 2156 |
| Fibrosis (LSM ≥ 10.0 kPa) | Waist circumference | 0.640 | 0.678 | 0.016 | 2156 |
| Fibrosis (LSM ≥ 10.0 kPa) | Fasting plasma glucose | 0.640 | 0.591 | 0.064 | 2156 |
| Fibrosis (LSM ≥ 8.0 kPa) | ALT/AST ratio | 0.649 | 0.528 | <0.001 | 2156 |
| Fibrosis (LSM ≥ 8.0 kPa) | Triglycerides | 0.649 | 0.508 | <0.001 | 2156 |
| Fibrosis (LSM ≥ 8.0 kPa) | TyG index | 0.649 | 0.526 | <0.001 | 2156 |
| Fibrosis (LSM ≥ 8.0 kPa) | Fasting plasma glucose | 0.649 | 0.565 | <0.001 | 2156 |
| Fibrosis (LSM ≥ 8.0 kPa) | BMI | 0.649 | 0.671 | 0.033 | 2156 |
| Fibrosis (LSM ≥ 8.0 kPa) | Waist circumference | 0.649 | 0.673 | 0.040 | 2156 |
| Steatosis (CAP ≥ 274 dB/m) | Waist circumference | 0.532 | 0.483 | 0.084 | 2156 |
| Steatosis (CAP ≥ 274 dB/m) | BMI | 0.532 | 0.498 | 0.130 | 2156 |
| Steatosis (CAP ≥ 274 dB/m) | ALT/AST ratio | 0.532 | 0.569 | 0.229 | 2156 |
| Steatosis (CAP ≥ 274 dB/m) | Fasting plasma glucose | 0.532 | 0.517 | 0.739 | 2156 |
| Steatosis (CAP ≥ 274 dB/m) | Triglycerides | 0.532 | 0.526 | 0.886 | 2156 |
| Steatosis (CAP ≥ 274 dB/m) | TyG index | 0.532 | 0.533 | 0.987 | 2156 |
| Steatosis (CAP ≥ 290 dB/m) | Waist circumference | 0.575 | 0.520 | 0.002 | 2156 |
| Steatosis (CAP ≥ 290 dB/m) | Triglycerides | 0.575 | 0.505 | 0.003 | 2156 |
| Steatosis (CAP ≥ 290 dB/m) | TyG index | 0.575 | 0.520 | 0.022 | 2156 |
| Steatosis (CAP ≥ 290 dB/m) | BMI | 0.575 | 0.543 | 0.026 | 2156 |
| Steatosis (CAP ≥ 290 dB/m) | Fasting plasma glucose | 0.575 | 0.542 | 0.214 | 2156 |
| Steatosis (CAP ≥ 290 dB/m) | ALT/AST ratio | 0.575 | 0.588 | 0.472 | 2156 |

*Interpretation guide: a smaller DeLong P value indicates a statistically significant AUC difference between the ZJU index and the comparator for the specified outcome.*

*Recommended use in the revision: cite this table in the response to the reviewer who requested more comparisons to evaluate the strengths and limitations of the ZJU index.*
